# Highly efficient decomposition of ammonia using high-entropy alloy catalysts

**DOI:** 10.1038/s41467-019-11848-9

**Published:** 2019-09-05

**Authors:** Pengfei Xie, Yonggang Yao, Zhennan Huang, Zhenyu Liu, Junlei Zhang, Tangyuan Li, Guofeng Wang, Reza Shahbazian-Yassar, Liangbing Hu, Chao Wang

**Affiliations:** 1 Department of Chemical and Biomolecular Engineering, Johns Hopkins University, Baltimore, MD 21218 USA; 2 Department of Materials Science and Engineering, University of Maryland, College Park, MD 20742 USA; 3 Department of Mechanical and Industrial Engineering, University of Illinois, Chicago, IL 60607 USA; 4 Department of Mechanical Engineering & Materials Science, University of Pittsburgh, Pittsburgh, PA 15261 USA

**Keywords:** Heterogeneous catalysis, Hydrogen storage, Nanoparticles, Synthesis and processing

## Abstract

Ammonia represents a promising liquid fuel for hydrogen storage, but its large-scale application is limited by the need for precious metal ruthenium (Ru) as catalyst. Here we report on highly efficient ammonia decomposition using novel high-entropy alloy (HEA) catalysts made of earth abundant elements. Quinary CoMoFeNiCu nanoparticles are synthesized in a single solid-solution phase with robust control over the Co/Mo atomic ratio, including those ratios considered to be immiscible according to the Co-Mo bimetallic phase diagram. These HEA nanoparticles demonstrate substantially enhanced catalytic activity and stability for ammonia decomposition, with improvement factors achieving >20 versus Ru catalysts. Catalytic activity of HEA nanoparticles is robustly tunable by varying the Co/Mo ratio, allowing for the optimization of surface property to maximize the reactivity under different reaction conditions. Our work highlights the great potential of HEAs for catalyzing chemical transformation and energy conversion reactions.

## Abstract (web summary)

Alloys are important materials for catalysis but are usually limited by miscibility gaps present in their phase diagrams. Here the authors break this limitation by developing high-entropy alloy catalysts made of five earth-abundant elements and demonstrate great catalytic enhancements for ammonia decomposition.

## Introduction

**Fig. 1:**
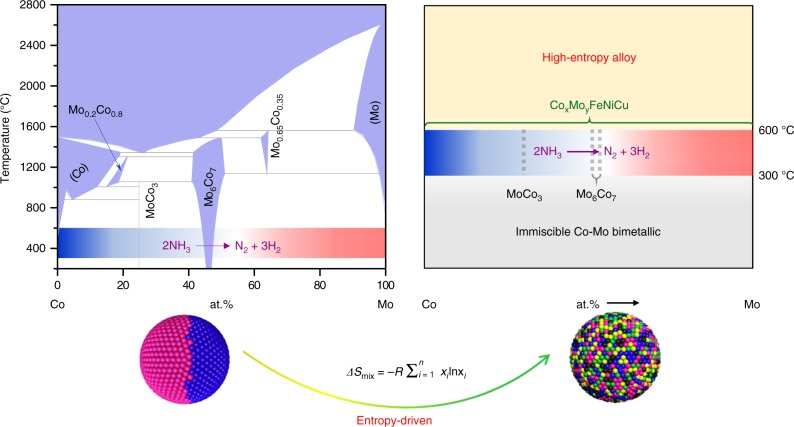
HEA catalysts breaking the miscibility limitation of conventional binary alloys. The phase diagram of bimetallic Co-Mo (left) is reprinted from Wang et al.^51^, with permission from Elsevier

The ammonia (NH_3_) decomposition reaction has received increasing attention for the potential use of NH_3_ as a hydrogen storage medium^1,2^. NH_3_ can be readily liquefied at a mild pressure of ~8 bar at room temperature, giving rise to an energy density of 4.25 kWh/L. Ruthenium (Ru) has been known as the most active metal for catalyzing the decomposition of ammonia, but its large-scale application is limited due to the scarcity and high cost of this precious metal^3–5^. Literature efforts have thus turned to alloys of earth-abundant elements^5–9^. In particular, bimetallic Co-Mo has been shown to be promising for ammonia decomposition, as rationalized by the computational interpolation of adsorption properties on the alloy surfaces with mixed sites^9,10^. However, the functional tuning and catalytic activity of Co-Mo catalysts are largely constrained by the large miscibility gap present in the phase diagram of this binary alloy (Fig. 1), and the experimental studies have primarily been limited to the catalysts with elemental ratio of Co/Mo around one^11–15^. How to overcome this hurdle is of great interest for both fundamental understanding of alloy catalysts and pratical improvement of the performance of earth-abundant materials in energy-conversion systems.

Here we report on a new class of high-entropy alloy (HEA) catalysts made of earth-abundant elements for highly efficient decomposition of ammonia. Enabled by a carbothermal shock technique, we have been able to grow HEA nanoparticles with five metals incorporated into a single solid-solution phase. We demonstrate breaking the miscibility limitation in bimetallic Co-Mo alloys by robustly tuning the Co/Mo element ratio in CoMoFeNiCu HEA nanoparticles (Fig. 1). These HEA nanoparticles are subjected to systematic and comprehensive catalytic studies for the NH_3_ decomposition and compared to bimetallic Co-Mo and monometallic Ru catalysts. A series of characterization techniques including atomically resolved scanning transmission electron microscopy (STEM), X-ray photoemission spectroscopy (XPS), and surface-specific temperature programmed desorption of nitrogen (nitrogen TPD) is employed to evaluate the structure-property relationship of the HEA catalysts. The obtained knowledge is further integrated with the measured reaction kinetics and simulated atomic structures of the HEA catalysts to understand the catalytic enhancement mechanisms.

## Results

### Synthesis and characterization of HEA nanoparticles

**Fig. 2:**
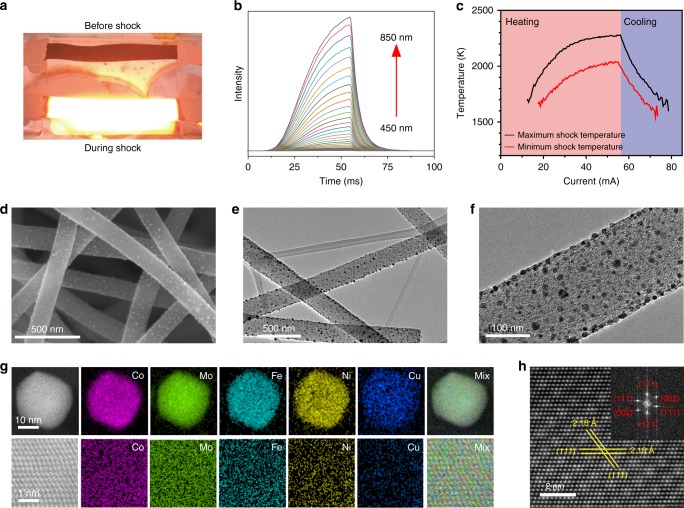
High-entropy alloy CoMoFeCoNi (HEA-Co_*x*_Mo_*y*_) nanoparticles. **a** Digital images of the samples before and during the thermal shock synthesis. **b** Intensity of the emitted light from the shock at different wavelengths. **c** Typical temperature profiles during the thermal shocks as determined from the emission intensities. **d** Representative SEM and **e**, **f** TEM images of the obtained HEA nanoparticles well dispersed on carbon nanofibers (CNFs). **g** STEM-based elemental maps of the HEA-Co_25_Mo_45_ nanoparticles at low- (upper panel) and high- (lower panel) resolutions. **h** High-resolution HAADF-STEM image with the fast Fourier transform (FFT) pattern indicating an fcc crystal structure

The CoMoFeNiCu HEA nanoparticles were synthesized by employing flash heating and cooling of metal precursors on oxygenated carbon supports, with the temperature going up to 2000–2300 K, the temperature ramping rate on the order of 10^5^ K s^−1^ and the duration of shocks as short as ~55 ms (Fig. 2a–c)^16^. Under such conditions, the high temperature induced rapid thermal-decomposition of the precursors, forming small liquid droplets of multimetallic solutions. The subsequent rapid cooling enabled crystallization of these liquid droplets into uniform and homogeneous alloy nanoparticles without being subjected to aggregation/agglomeration, element segregation or phase separation. Five types of HEA nanoparticles with the general composition Co_*x*_Mo_*y*_Fe_10_Ni_10_Cu_10_ (*x* + *y* = 70) were prepared by using this method, in which the atomic ratio of Co/Mo was controlled to be 15/55, 25/45, 35/35, 45/25, and 55/15 by varying the loading of precursors, the overall loading of metals in each catalyst was controlled at 10 wt% during synthesis. These nanoparticles are denoted as HEA-Co_*x*_Mo_*y*_ (e.g., HEA-Co_15_Mo_55_ for Co_15_Mo_55_Fe_10_Ni_10_Cu_10_) in the following discussion. Bimetallic Co-Mo and monometallic Ru nanoparticles were also synthesized using the same method with similar metal loadings, which served as control in this study.

Figure 2d shows representative scanning electron microscopy (SEM) images of the C_25_Mo_45_Fe_10_Ni_10_Cu_10_ nanoparticles uniformly dispersed on the carbon nanofibers (see more images in Supplementary Fig. 1). The spacing between neighboring nanoparticles varies from ~10 to ~100 nm. The average particle size is measured to be ~22 nm from the transmission electron microscopy (TEM) images (Fig. 2e, f; also see more images in Supplementary Fig. 2). Low- and high-magnification elemental maps depict homogeneous distribution of all the five elements throughout the C_25_Mo_45_Fe_10_Ni_10_Cu_10_ nanoparticles (Fig. 2g and Supplementary Fig. 3), and similar observations were also obtained on the HEA nanoparticles of different compositions (Supplementary Figs. 4–7). In contrast, the bimetallic Co_36_Mo_64_ nanoparticles (corresponding to Co/Mo = 25/45) show separated Co- and Mo-rich phases (Supplementary Fig. 8), as this composition falls into the miscibility gap in the corresponding binary phase diagram (Fig. 1). Atomically resolved high-angle annular dark-field scanning TEM (HAADF-STEM) imaging and the corresponding fast Fourier transform analysis reveal a face-centered cubic (*fcc*) phase for the HEA nanoparticles, with the inter-plane spacing measured to be 2.18 Å for the (111) lattice fringes (Fig. 2h).

**Fig. 3:**
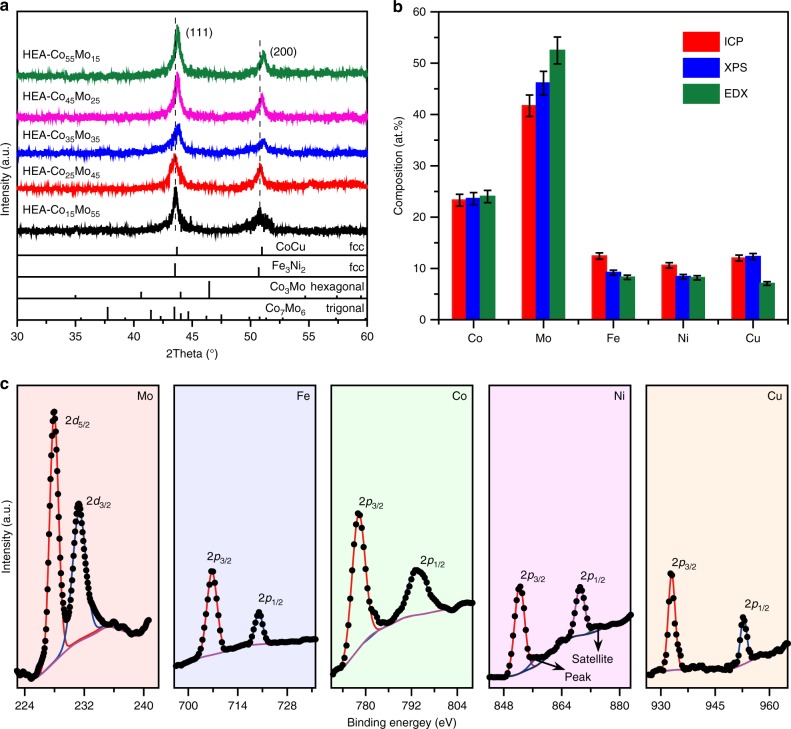
Characterization of the HEA-Co_*x*_Mo_*y*_ nanoparticles. **a** XRD patterns of the HEA nanoparticles supported on CNFs in comparison to the bimetallic standards of CoCu (PDF# 50–1452), Fe_3_Ni_2_ alloy (PDF# 65–5131), Co_3_Mo (JCDPS no. 29–0488), and Co_7_Mo_6_ (JCDPS no. 29–0489). **b** Comparison of the composition analyses for the HEA-Co_25_Mo_45_ nanoparticles based on three different techniques: ICP-MS, XPS, and EDX, verifying the alloy homogeneity in the HEA nanoparticles. Error bars denote SDs. **c** XPS spectra collected at the Mo 3*d*, Fe 2*p*, Co 2*p*, Ni 2*p,* and Cu 2*p* edges for the HEA-Co_25_Mo_45_ nanoparticles

Crystal structure of the HEA nanoparticles was further confirmed by X-ray diffraction (XRD) analysis. In the XRD patterns (Fig. 3a), the HEA nanoparticles typically exhibit two peaks at around 44^o^ and 51^o^, which can be assigned to the (111) and (200) planes of a fcc crystal, A small upshift of the peak position is discernible as the Co/Mo ratio increases, but merely by ~0.5^o^ for the (111) peak from C_15_Mo_55_Fe_10_Ni_10_Cu_10_ to C_55_Mo_15_Fe_10_Ni_10_Cu_10_, indicating rather small differences in lattice strain among the HEA nanoparticles of various compositions. It is noticed that only two crystal phases are known for bimetallic Co-Mo alloys, i.e., Co_3_Mo with a hexagonal structure (*P6*_*3*_*/mmc*) and Co_7_Mo_6_ with a trigonal structure (*R-3m*). The XRD patterns recorded for the HEA-Co_*x*_Mo_*y*_ nanoparticles do not match either one of these two phases (Fig. 3a), but are more in line with those of the bimetallic alloys in *fcc* phases albeit with shifted peak positions (Fig. 3a and Supplementary Fig. 8). This observation is consistent with the features of high-entropy alloys and confirms that the derived nanoparticles are in a single *fcc* solid-solution phase^17–19^. The rather low peak intensities can be owing to the much smaller particle sizes than those previously reported HEAs^20^.

A combination of inductively coupled plasma-mass spectrometer (ICP-MS), X-ray photoemission spectroscopy (XPS) and energy dispersive X-ray spectroscopy (EDX) was employed to analyze the element compositions of the HEA nanoparticles. Among these three techniques, ICP (Table 1) and EDX provide composition information for the bulk of the nanoparticles, and average over the whole catalyst, whereas XPS is more sensitive to the surface region (~1 nm in depth estimated from the beam energy of Al K*α*). The three methods generated quite consistent results for the alloy compositions (Fig. 3b), which is consistent with the homogeneous alloy nature as revealed by the STEM-based element mapping and excludes the occurrence of surface segregation or phase separation. The XPS analysis also shows that all the constituting elements of the HEA nanoparticles are in metallic states. Figure 3c presents representative XPS spectra recorded at the 3*d* edge of Mo and 2*p* edges of Co, Fe, Ni, and Cu (see more spectra at the other edges in Supplementary Fig. 9). For example, the two peaks associated with the Mo 3*d*_5/2_ and 3*d*_3/2_ doublet are located at 228.0 and 231.2 eV, respectively, while the Co 2*p*_3/2_ and 2*p*_1/2_ peaks are at 778.2 and 793.3 eV, respectively. Both of these two sets of binding energies, as well as those for Fe, Ni, and Cu, are in line with the established values for pure metals^21^. Charge transfer between the metals, or ligand effect^22,23^, thus may not be significant in the HEA-Co_*x*_Mo_*y*_ nanoparticles.

**Table 1:** List of HEA-Co_x_Mo_y_ catalysts employed in this study

Samples	Co	Mo	Fe	Ni	Cu	Metal loading^a^	S_BET_^b^
	%	%	%	%	%	wt.%	m^2^/g
HEA-Co_15_Mo_55_	14.3	51.8	12.6	11.2	10.1	8.2	150
HEA-Co_25_Mo_45_	23.3	41.7	12.4	10.6	12.0	7.8	157
HEA-Co_35_Mo_35_	33.5	32.3	12.0	10.8	11.4	8.3	160
HEA-Co_45_Mo_25_	42.9	23.5	11.7	11.5	10.4	8.8	148
HEA-Co_55_Mo_15_	52.8	13.2	11.9	10.8	11.3	9.3	153

^a^ The overall weight percentages of metals in each catalyst as determined by ICP-MS analyses

^b^ Estimated from the N_2_ adsorption isotherms according to the BET theory

### Catalytic studies for ammonia decomposition

**Fig. 4:**
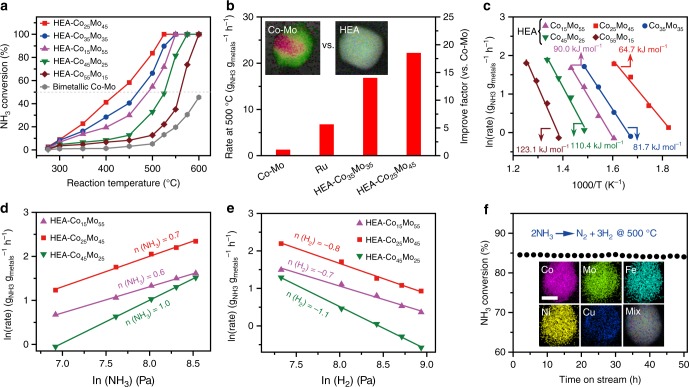
Performance of HEA-Co_*x*_Mo_*y*_ catalysts for NH_3_ (5 vol%) decomposition. **a** NH_3_ conversions over different HEA-Co_*x*_Mo_*y*_ nanoparticles and bimetallic Co-Mo (Co/Mo = 25/45) depending on the reaction temperature (Space velocity = 36 L g_cata_^−1^ h−^1^). **b** Comparison of reaction rates measured in the kinetic regime among bimetallic Co-Mo, Ru, and HEA-Co_*x*_Mo_*y*_ catalysts (*T* = 500 ^o^C). Inset: element maps for the bimetallic Co-Mo (Co/Mo = 25/45) and HEA-Co_25_Mo_45_ catalysts. **c** Arrhenius plots for NH_3_ decomposition on the HEA-Co_*x*_Mo_*y*_ catalysts, showing different apparent activation energies (*E*_app_). **d**, **e** Reaction orders of NH_3_ and H_2_ determined for the NH_3_ decomposition reaction at 425 ^o^C on the HEA-Co_*x*_Mo_*y*_ catalysts. **f** Stability test performed at 500 ^o^C for the HEA-Co_25_Mo_45_ catalyst. Inset: element map of the catalyst after the stability test; scale bar = 10 nm

The HEA-Co_*x*_Mo_*y*_ nanoparticles supported on carbon nanofibers were directly applied as catalysts for the ammonia decomposition reaction and compared to the bimetallic Co-Mo with the ratio of 25/45 (~20 nm in particle size, Supplementary Fig. 10, see the elemental maps of bimetallic Co-Mo with other ratios in Supplementary Fig. 11) and monometallic Ru (~2–3 nm, Supplementary Fig. 12) catalysts with similar metal loadings and same substrates. The catalytic activity was systematically measured at 250–600 ^o^C using a plug flow reactor and 5 vol% NH_3_ as the feeding gas. The bare CNF substrate was also measured and confirmed to be inactive for NH_3_ decomposition (Supplementary Fig. 13). Figure 4a summarizes the measured NH_3_ conversion as a function of temperature at a gas hourly space velocity (GHSV) of 36 L g_cat_^−1^ h^−1^. For all the HEA-Co_*x*_Mo_*y*_ catalysts, the reaction has an onset temperature of ~300 ^o^C, and the NH_3_ conversion increases with the reaction temperature. Between 300 and 500 ^o^C, the NH_3_ conversion follows the order HEA-Co_25_Mo_45_ > HEA-Co_35_Mo_35_ > HEA-Co_15_Mo_55_ > HEA-Co_45_Mo_25_ > HEA-Co_55_Mo_15_, reaching 50% of conversion (T_50_) at ca. 422, 466, 488, 523, and 558 ^o^C, respectively. The reaction reaches saturation (100% conversion) at ~525 ^o^C on HEA-Co_25_Mo_45_, as compared to ~600 ^o^C for HEA-Co_55_Mo_15_. Most of the HEA catalysts are much more active than the bimetallic Co-Mo and monometallic Ru, with the latter two giving NH_3_ conversions of only 46 and 73% at 600 ^o^C (Supplementary Fig. 14). Figure 4b compares the reaction rate measured at 500 ^o^C among the two most active HEA catalysts, bimetallic Co-Mo and monometallic Ru. The HEA-Co_35_Mo_35_ and -Co_25_Mo_45_ catalysts reach a mass-specific rate of 16.7 and 22.1 g_NH3_ g_metals_^−1^ h^−1^ at 500 ^o^C, representing improvement factors of ~14 and ~19 versus Co-Mo, respectively. Moreover, higher mass-specific activities were obtained with the HEA catalysts of reduced particle sizes (Supplementary Fig. 15). Compared to the precious metal Ru, the Co_25_Mo_45_Fe_10_Ni_10_Cu_10_ catalyst achieves an improvement factor of ~3 versus Ru. To take the different particle sizes into account, HEA-Co_25_Mo_45_ achieved an area-specific reaction rate of 0.74 g_NH3_ m^−2^ h^−1^, representing improvement factors of ~24 versus bimetallic Co-Mo and ~19 versus Ru (Supplementary Fig. 16). The superior performance of the HEA catalysts for NH_3_ decomposition is further revealed by a comprehensive comparison to the literature results with similar reaction conditions (Table 2).

**Table 2:** Comparison of the catalytic performance for NH_3_ decomposition of different catalysts

Catalysts	Metals	NH_3_	T	GHSV	Conversion	TOF^a^	Ref
	(wt%)	(vol%)	(°C)	(mL g_cat_^−1^ h^−1^)	(%)	(h^−1^)	
Ru/SiO_2_	10	100	500	30,000	64	2283	2
Ru/Al_2_O_3_	10	100	500	30,000	58	2069	26
Ru/CNTs	4.8	100	500	30,000	84	6241	5
Ru/TiO_2_	4.8	100	500	30,000	64	11,096	27
Ru/MgO	5	100	500	30,000	41	8189	24
Co(en)_3_MoO_4_	38.8	100	500	36,000	12	294	14
Co_3_Mo_3_N	97	50	500	22,500	33	409	12
CoMo/SiO_2_	5	100	500	36,000	14	1108	47
FeMo/La-Al_2_O_3_	10	100	500	46,000	8	1213	48
Ni_2_Mo_3_N	97	100	500	21,600	29	204	49
Li_2_NH-Fe_2_N	81.2	100	500	60,000	38.1	1179	50
Li_2_NH-Mn_2_N	81	100	500	60,000	59.1	2735	50
HEA-Co_25_Mo_45_	7.8	5	500	36,000	84	1571	This work
HEA-Co_35_Mo_35_	8.3	5	500	36,000	67	1128	This work
HEA-Co_45_Mo_25_	8.8	100	500	36,000	64.5	19,633	This work
HEA-Co_55_Mo_15_	9.3	100	500	36,000	100	25,209	This work

^a^ Obtained by normalizing the reported reaction rates with the areal densities of surface atoms estimated from the particle sizes

Kinetic studies were also performed on the HEA catalysts with varying reaction temperature and gas composition. Figure 4c presents the Arrhenius plots of the reaction rate in dependence of temperature. The determined apparent activation energy (*E*_app_) varies with the Co/Mo ratio, with the trend being consistent with that for the NH_3_ conversion, namely from the lowest 64.7 kJ mol^−1^ for the most active HEA-Co_25_Mo_45_ to 123.1 kJ mol^−1^ for the least active HEA-Co_55_Mo_15_ catalyst. The activation energy measured here for HEA-Co_25_Mo_45_ is comparable to those previously reported for Ru-based catalysts (57.8–83.7 kJ mol^−1^)^24–27^. The determined reaction order of NH_3_ at 425 ^o^C increases with the Co/Mo ratio, varying from 0.6 for HEA-Co_15_Mo_55_ to 1.0 for HEA-Co_45_Mo_25_ (Fig. 4d). This indicates that the activation of NH_3_ is more difficult on the Co-rich catalysts. On the contrary, the H_2_ order (at 425 ^o^C) decreases from −0.7 for HEA-Co_15_Mo_55_ to −1.1 for HEA-Co_45_Mo_25_, suggesting a less inhibition effect of H_2_ on the Mo-rich catalysts (Fig. 4e)^28^. The partial pressure of N_2_ was found to have little effect on the reaction rate (Supplementary Fig. 17), which is consistent with the previous conclusion drawn from Ru-based catalysts^29^.

In addition to the enhanced catalytic activities, the HEA-Co_*x*_Mo_*y*_ catalysts were further demonstrated to be highly stable under the reaction conditions for NH_3_ decomposition. Figure 4f presents the NH_3_ conversion recorded on Co_25_Mo_45_Fe_10_Ni_10_Cu_10_ over the course of continuous operation at 500 ^o^C. The degradation in catalytic activity was negligible after ~50 h. The catalyst collected after this prolonged durability test was characterized by element mapping (see the insert in Fig. 4f) and XPS (Supplementary Fig. 18), with nearly no change found in alloy homogeneity or surface composition. A small amount (~1.4 at.%) of *N* was detected on the used catalyst (Supplementary Fig. 19), but much less than what (e.g., >10% for Mo_3_Co_3_N) one would expect for the formation of nitrides throughout the nanoparticles. It is likely that the bulk of the HEA nanoparticles were not subjected to nitridation during the NH_3_ decomposition reaction. The durable performance of the HEA catalysts is in line with the reported high thermal^30^ and chemical^31,32^ stabilities of HEAs.

### Adsorption property and catalytic descriptor

The reaction mechanism of ammonia decomposition has been intensively investigated in both experimental and computational studies^1,6,8,25^. It is suggested that the recombinative desorption of nitrogen is the rate-determining step on Fe, Co, and Ni, while the kinetics is limited by scission of the first N-H bond on Rh, Ir, Pd, Pt, and Cu catalysts^4^, although some debates are still present in the literature^33,34^. Nevertheless, it is generally accepted that the binding strength of nitrogen is a good descriptor for ammonia decomposition catalysts, which largely determines both the stability of adsorbing intermediates (e.g., *N and *NH_*x*_) and activation energy of the rate-determining step^6,10,12^. In order to interpret the observed kinetic performance, we have performed temperature programmed desorption (TPD) of pre-adsorbed atomic nitrogen (2*N → N_2_) to evaluate the adsorption properties of the HEA catalysts^35–38^.

**Fig. 5:**
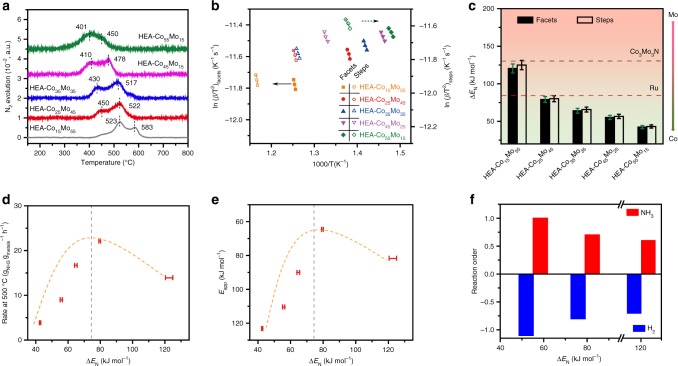
Surface adsorption properties of HEA-Co_*x*_Mo_*y*_. **a** Nitrogen TPD profiles recorded with a temperature ramping rate of 10 ^o^C min^−1^. **b**, **c** Estimation of the nitrogen adsorption energies (Δ*E*_*N*_) for the HEA-Co_*x*_Mo_*y*_ catalysts by using the plots of Ln(*β*/*T*^2^) ~ 1/*T* for the nitrogen TPD profiles (*β* = 10, 20, and 30 ^o^C min^−1^ represents the ramping rates used for the TPD measurements). **d**, **e** Correlations between Δ*E*_*N*_ and catalytic activities (**d**) and *E*_*app*_ (**e**) showing a volcano-type behavior for the HEA-Co_*x*_Mo_*y*_ catalysts, with HEA-Co_25_Mo_45_ being close to the peak position. **f** Plots of the NH_3_ and H_2_ reaction orders (at 425 ^o^C) versus the nitrogen adsorption energies. Error bars denote SDs

Figure 5a shows the nitrogen TPD patterns recorded at a ramping rate of 10 ^o^C min^−1^. Two distinct desorption peaks are consistently observed on all the HEA-Co_*x*_Mo_*y*_ catalysts in the temperature range of 400–600 ^o^C. These features resemble the previously reported results for Ru^35,36^ and Fe^37,38^ surfaces, and can be assigned to the recombinative desorption of nitrogen on ordered facets (such as (111) and (100) for the first peak) and undercoordinated sites (steps, edges, defects, etc. for the second peak), with the latter having stronger binding to nitrogen and thereby higher desorption temperatures due to the lower coordination numbers. A clear trend is established for the HEA catalysts of different compositions, with the desorption temperatures (both onset and peak positions) rising at decreasing Co/Mo ratios, suggesting the continuous tuning of nitrogen binding strength by varying the HEA composition. According to the TPD theorem^39,40^, we have conducted additional measurements at different ramping rates (*β* = 20 and 30 ^o^C min^−1^) to establish the plots of ln(*β*/*T*_*m*_^2^) versus 1/*T*_*m*_ (*T*_*m*_ is the peak position), in which the slopes are used to estimate the recombinative desorption energy of nitrogen (Δ*E*_N_) (Fig. 5b and Supplementary Fig. 20). Figure 5c summarizes the derived values of Δ*E*_N_ for the different HEA-Co_*x*_Mo_*y*_ catalysts. As expected with the observations from the TPD patterns, the catalysts with higher Co/Mo ratios have lower Δ*E*_N_, ranging from ~121–125 kJ mol^−1^ for HEA-Co_15_Mo_55_ to ~42–44 kJ mol^−1^ for HEA-Co_55_Mo_15_. It is noticed that the most active HEA-Co_25_Mo_45_ catalyst has a Δ*E*_N_ value of ~79 kJ/mol, which is very close to the Δ*E*_N_ (84 kJ mol^−1^)^24^ of Ru, explaining the high and comparable catalytic activities of the HEA-Co_*x*_Mo_*y*_ to Ru-based catalysts. HEA-Co_35_Mo_35_ has the same Co/Mo ratio as Co_3_Mo_3_N but binds to nitrogen much less strongly (Fig. 5c). This difference (~65 kJ mol^−1^) can be ascribed to the presence of other weakly binding metals (Fe, Ni, and Cu) in and on the surface of the HEA catalysts.

**Fig. 6:**
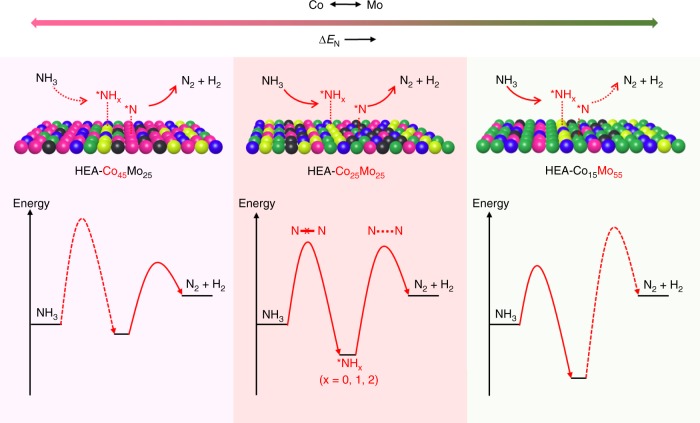
Schematic illustration of the rate-limiting factors in NH_3_ decomposition. The rate-limiting factors are labeled with dash lines in the lower panel. On Co-rich surface (left), the rate is limited by activation or dehydrogenation of NH_3_; on Mo-rich surface, the rate is limited by the recombinative desorption of *N; Balance for these two steps is reached on an intermediate composition (HEA-Co_25_Mo_45_)

With the estimated nitrogen adsorption energies, we are able to interpret the correlation between composition and catalytic performance of the HEA-Co_*x*_Mo_*y*_ catalysts. Figure 5d, e present the plots of NH_3_ decomposition rate (at 500 ^o^C) and activation energy versus Δ*E*_N_. Both plots exhibit a volcano-type behavior with HEA-Co_25_Mo_45_ being close to the peak position. According to the Sabatier principle, we can infer that, on the left side of this peak, the catalysts (more Co-rich) bind to N too weakly and thus gives rise to rather higher kinetic barriers for dehydrogenation (NH_3_ → *NH_2_ → *NH → *N), whereas on the right side (more Mo-rich) the binding is too strong for N to recombine and desorb from the surface (2*N → N_2_), and a tradeoff between these two factors gives rise to an optimal, intermediate binding energy for the reaction (Fig. 6). The finding here can be further correlated to the dependence of reaction orders on Δ*E*_N_. As shown in Fig. 5f, the catalysts with weaker N binding energy has higher NH_3_ order, suggesting that the kinetics of NH_3_ decomposition is more limited by NH_3_ activation, or probably dehydrogenation of the first N-H bond, on the HEA catalysts of higher Co contents. Since the binding strengths of nitrogenous species (*NH_*x*_, *x* = 0, 1, 2, and 3) likely scale linearly with each other^41–43^, we can expect that the *NH_*x*_ species bind less strongly on the more Co-rich HEA catalysts, which is also in line with the stronger hydrogen inhibition effect found for these catalysts (Fig. 5f).

**Fig. 7:**
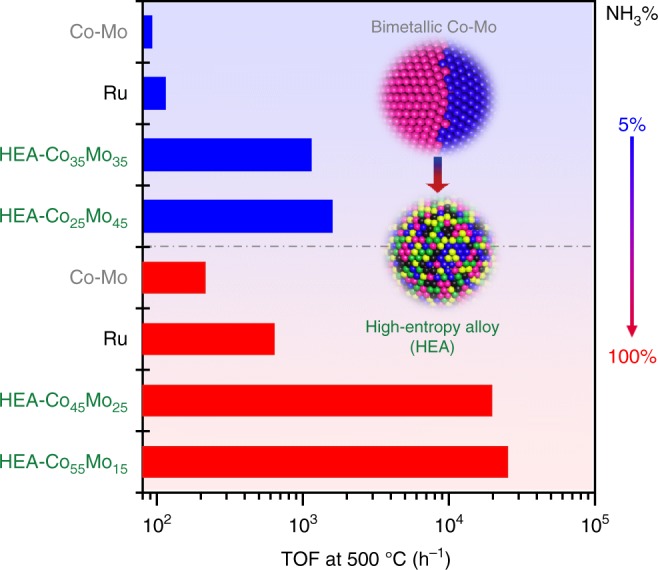
NH_3_ decomposition at different concentrations. The composition of HEA-Co_*x*_Mo_*y*_ catalysts were robustly tuned to optimize the surface properties for different reaction conditions (e.g., 5 vs. 100 vol% NH_3_)

From the above discussion, we can attribute the high catalytic activity of NH_3_ decomposition achieved with the HEA catalysts to the robust tuning of surface adsorption properties, as enabled by the continuous varying of alloy composition (Co/Mo ratio). Noticeably, the optimal value of Δ*E*_N_ ~ 79 kJ mol^−1^ identified here is consistent with the value (~74 kJ mol^−1^) predicted from density functional theory calculations for the given reaction condition, i.e., 5 vol% of NH_3_^12^. We also notice that, for the optimal design of practical reactors, it is desirable to have cascaded catalyst layers with gradually changing nitrogen binding strengths, so that the surface property can be optimized to match the varying concentration of NH_3_ along the flow^44^. We show here that the HEA catalysts can be readily tailored to achieve such a design by simply varying the Co/Mo ratios. For example, HEA-Co_55_Mo_15_ have been identified to be the best-performing catalyst for the decomposition of pure NH_3_, which gives rise to catalytic activity improvement factors of ~118 versus bimetallic Co-Mo and ~40 versus Ru (Fig. 7 and Supplementary Fig. 21). The even more substantial catalytic enhancements found here (than the results presented above for 5 vol% of NH_3_) can be well understood in such a way: the Δ*E*_N_ value of HEA-Co_55_Mo_15_ (~42 kJ mol^−1^, according to Fig. 5c) is very close to the optimal (~39 kJ mol^−1^) predicted for the reaction at 100 vol% of NH_3_^12^, whereas the values of previous Co_3_Mo_3_N and Ru catalysts are quite away from this optimal point.

### Atomistic modeling of HEA nanoparticles

The correlations among composition, adsorption property and catalytic performance discussed above point to a scaling relationship between Δ*E*_N_ and the elemental ratio of Co/Mo (Fig. 5c), which governs the catalytic activity of the HEA catalysts. This relationship can be ascribed to the presence of well mixed Co-Mo sites on the surface with the ratio being consistent with the bulk compositions (Fig. 3b). We have used atomistic models to elucidate this surface site mixing mechanism in the HEA catalysts.

**Fig. 8:**
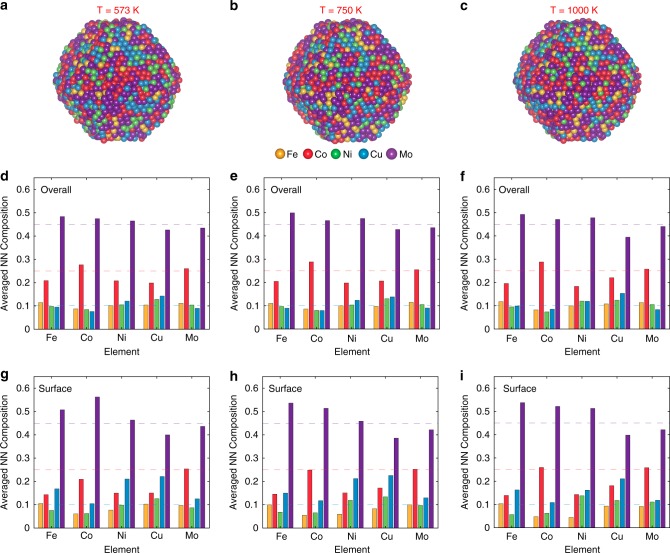
Atomic structure of HEA catalysts. Atomistic model of HEA-Co_25_Mo_45_ nanoparticle predicted by using MC simulations at different temperatures: **a** 573 K; **b** 750 K; and **c** 1000 K. The composition of the first-nearest neighbor lattice sites around a specific element type averaged over (**d**–**f**) all atoms and (**g**–**i**) surface atoms in the corresponding nanoparticle. The dashed lines represent the nominal composition of the nanoparticle (Co_0.25_Mo_0.45_Fe_0.1_Ni_0.1_Cu_0.1_)

HEA-Co_25_Mo_45_ (Co_25_M_45_Fe_10_Ni_10_Cu_10_) was chosen as the example and modeled in a cuboctahedral shape with FCC lattice. From the survey of those bulk multi-component alloys to form solid-solution HEAs, it has been found that the formation of HEA most often requires: the parameter gauging the atomic size difference *δ* ≤ 6.6% and the enthalpy of formation ${\mathrm{ - 11}}{\mathrm{.6}} \,\,< \,\, \Delta H_{{\mathrm{mix}}} \,\, < \,\, {\mathrm{3}}{\mathrm{.2}}\left( {{\mathrm{kJmol}}^{{\mathrm{ - 1}}}} \right)$^45,46^. In this work, we have calculated that HEA-Co_25_Mo_45_ has $\delta = {\mathrm{5.4}} \%$, $\Delta H_{{\mathrm{mix}}} = {\mathrm{0}}{\mathrm{.65}}\left( {{\mathrm{kJmol}}^{{\mathrm{ - 1}}}} \right)$, and $\Delta S_{{\mathrm{mix}}} = {\mathrm{11}}{\mathrm{.6}}\left( {{\mathrm{J}}\left( {{\mathrm{Kmol}}} \right)^{{\mathrm{ - 1}}}} \right)$. Hence, we predict that HEA-Co_25_Mo_45_ is likely to form solid-solution HEAs under the ammonia decomposition reaction conditions. The five elements are initially randomly assigned to each lattice site according to the nominal composition. To investigate the phase stability of the HEA nanoparticles, Monte Carlo (MC) simulations are performed at three different temperatures (i.e., 573, 750, and 1000 K). The atomic structures after 10 million MC steps are shown in Fig. 8a–c. All the resulting structures show random distribution of the five elements, and no apparent chemical ordering such as the segregation of single elements or formation of intermetallic phases was observed, confirming the formation of HEA under the given conditions. This finding is further corroborated by statistical analysis of the averaged composition of the nearest-neighbor lattice sites (with a cutoff radius of 3.0 Å) for a given element (Fig. 8d–i). For an ideal solid solution, this averaged composition should be equal to the nominal composition of the HEA nanoparticle, which are represented by the dash lines in Fig. 8d–i. Our results show that the nearest-neighbor (NN) compositions averaged over all the atoms only deviate slightly from the nominal composition throughout the three temperatures investigated, implying the absence of long-range chemical ordering. On the other hand, some degree of short-range ordering is predicted in the modeled nanoparticle: Fe, Co, and Ni atoms exhibit a relatively higher affinity around Mo atom, whereas there is a moderate repulsive interaction between Cu-Mo, Fe-Co, Co-Ni, and Co-Cu atom pairs. This short-range ordering is even prominent for surface atoms, as shown in Fig. 8g–i. Overall, our atomistic modeling reveals that the HEA-Co_*x*_Mo_*y*_ nanoparticles have a single solid solution phase with only slight short-range chemical ordering throughout the investigated temperature range. It also confirms the presence of well mixed surface sites on the HEA catalysts and suggests that the experimentally observed scaling relationship between the nanoparticle composition and the surface adsorption property can be understood via the surface site mixing mechanism^9,10^. It should be noted that our atomistic modeling results are consistent with the prediction based on simple thermodynamic calculations, confirming the tendency of the given quinary composition to form a single solid-solution phase. More importantly, the structural similarity at the different temperatures (from 573 to 1000 K) underlines the high thermal stability of the HEA nanoparticles, consistent with the observation from the catalytic durability studies (see Table 2)^47–51^.

## Discussion

We have developed advanced ammonia decomposition catalysts based on high-entropy alloys made of earth-abundant elements. The HEA CoMoFeNiCu nanoparticles enabled robust tuning of the Co/Mo ratio and breaking the miscibility limitation in conventional bimetallic Co-Mo catalysts. These novel catalysts achieved significantly enhanced catalytic activities and stabilities, with the improvement factors exceeding 20 times as compared to precious metal Ru, and even more so versus conventional Co-Mo catalysts. Their catalytic activities and kinetics were further found to exhibit a volcano-type behavior independent of the Co/Mo ratio, which was successfully interpreted by using the nitrogen adsorption energy as the descriptor and a mixed surface site mechanism derived from atomistic modeling. The robust tunability of alloy composition and surface adsorption property demonstrated on the HEA catalysts indicate great potential for their implementation in practical reactors with the catalytic performance optimized under various reaction conditions.

## Methods

### Synthesis

8 wt% polyacrylonitrile (PAN, Sigma Aldrich) in dimethylformamide (DMF, Sigma Aldrich) was used to make a polymer nanofiber network via electrospinning (voltage: 15 kV, distance: 10 cm, feed rate: 0.065 ml min^−1^, collected on a rotating drum at 40 rpm). The derived nanofibers were stabilized in air at 533 K for 6 h and then carbonized at 1173 K for 2 hours in argon using a tube furnace. The CNF films can be further thermally activated at 1023 K for 2 h in CO_2_ atmosphere to create surface defects for effective particle dispersion.

Typically, the individual metal salts (chloride salts, FeCl_3_, CoCl_2_, NiCl_2_, CuCl_2_, and MoCl_3_ from Sigma Aldrich) were dissolved in ethanol with five designed ratios of Co_*x*_Mo_*y*_Fe_10_Ni_10_Cu_10_ (*x* *+* *y* = 70, and the atomic ratio of Co/Mo was controlled to be 15/55, 25/45, 35/35, 45/25, and 55/15). The CNF films were suspended over the trench of two glass slides (2 cm gap) and then connected to two copper electrodes by silver paste for subsequent precursor solution loading. Then the salt precursor solution was dipped onto the CNF film with a precursor loading of 5 μmol cm^−2^. The samples were left to dry at room temperature.

The carbothermal shock process was performed through Joule heating of the precursor-loaded CNF films in an argon-filled glovebox. A Keithley SourceMeter (2425) was used as the electrical power source and the sweep function from Keithley was used to provide the thermal shock, in which the current (temperature), duration (thermal shock time), and cooling speed (ramp rate) of the electrical pulse can be adjusted. We chose a 55-ms electrical pulse to synthesize the high-entropy alloy nanoparticles.

We used a time-resolved pyrometer to measure the emitted light intensity from the sample and estimate the temperature during the thermal shock process. The emitted light was dispersed at a resolution of 6.5 nm mm^−1^ and then collected by a 32-channel photomultiplier tube array. The full spectrum can be integrated from the 32-channels and then fitted to the blackbody radiation equation to estimate the temperature.

The control sample, bimetallic Co-Mo supported on CNFs (Co/Mo = 15/55, 25/45, 35/35, 45/25, and 55/15), were prepared by the same method as HEA nanoparticles did. Typically, CoCl_2_ and MoCl_3_ were dissolved in ethanol with the atomic ratio of Co/Mo was 25/45 and total loading of 10 wt%. Then the salt precursor solution was dipped onto the CNF film with a precursor loading of 5 μmol cm^−2^. The samples were left to dry at room temperature, and then underwent the carbothermal shock process described as above.

Another control sample, Ru/CNF was prepared by incipient wetness impregnation. Basically, 50 mg carbon nanofibers were dispersed in ethanol, and sonicated for 1 h. Certain amount of RuCl_3_·xH_2_O was dissolved in ethanol (Nominal Ru loading is 10 wt%). Then these solutions were mixed together and sonicated for another 30 min. After that, the solvent was removed by using a rotary evaporator. The obtained solid was dried in vacuum and then calcined at 700 ^o^C in argon for 2 h.

### Characterization

X-ray diffraction (XRD) patterns were collected on a PANalytical X’Pert^3^ X-ray diffractometer equipped with a Cu K*α* radiation source (*λ* = 1.5406 Å). Nitrogen adsorption measurements were performed using a Micromeritics ASAP 2010 system with the samples degassed under vacuum at 300 °C for 5 h. Specific surface area (SSA) was calculated using the Brunauer-Emmett-Teller (BET) theory. Inductively coupled plasma-mass spectroscopy (ICP-MS) analysis was carried out using a PerkinElmer Elan DRC II Quadrupole system, for which the solutions were prepared by digesting the catalysts in aqua regia followed by dilution with 2% hydrochloric acid (HCl).

The microstructure and morphology of the HEA catalysts were characterized by using scanning electron microscopy (SEM, Hitachi SU-70 FEG-SEM at 10 kV) and transmission electron microscopy (TEM, JEOL 2100 F FEG TEM/STEM operated at 200 kV) imaging. High-angle annular dark-field (HAADF) STEM images and STEM-EDS mapping were acquired using a JEOL TEM/STEM ARM 200CF (equipped with an Oxford X-max 100TLE windowless X-ray detector) at a 22-mrad probe convergence angle and a 90-mrad inner-detector angle.

X-ray photoelectron spectroscopy (XPS) analysis was performed on a Thermo Fisher Scientific Escalab 250Xi spectrometer with Al K*α* radiation as the excitation source. Before measurements, the Co_x_Mo_y_-HEA catalysts were reduced in H_2_ at 600 ^o^C for 2 h. The adventitious carbonaceous C 1 s line (284.6 eV) was used to calibrate the binding energy (BE). The XPS spectra were deconvoluted using Gaussian-Lorentzian functions after the Shirley background subtraction, with the integrated peak areas being used to estimate the surface chemical compositions.

The nitrogen TPD experiments were carried out on a Micromeritics AutoChem II chemisorption analyzer equipped with an Omnistar MS detector. Typically, 30 mg of Co_x_Mo_y_-HEA catalysts were loaded in a U-type sample tube, reduced in H_2_ (30 mL min^−1^) at 600 ^o^C for 2 h, and then blown with He (30 mL min^−1^) for 1 h to remove the hydrogen adsorbed on the catalyst surface. After this, the flowing gas was switched to N_2_ (30 mL min^−1^) and kept at 600 ^o^C for 90 min, then cooled down to 100 ^o^C under N_2_ atmosphere. At 100 ^o^C, N_2_ was switched to He for removing the residual nitrogen on the catalyst surface. Finally, TPD measurement was conducted under He atmosphere at a ramping rate of 10 ^o^C min^−1^ and up to 800 ^o^C. Over this process, mass spectrum signals of He (4 amu) and N_2_ (28 amu) were monitored. For measuring combinative N_2_ desorption energy, three different ramping rates (10, 20, 30 ^o^C min^−1^) were used in a similar process.

### Catalytic studies

Catalytic decomposition of NH_3_ was conducted in a fixed-bed flow reactor at atmospheric pressure. Typically, 25 mg of catalyst was loaded into a quartz tube reactor (7 mm i.d.). The catalyst was heated to 600 ^o^C at a rate of 5 ^o^C min^−1^ under H_2_ (50 mL min^−1^) for 2 h and then purged by Helium (50 mL min^−1^) for 1 h. After this, the catalyst was cooled down to 275 ^o^C under He atmosphere. At 275 ^o^C, the gas flow was switched to 5 or 100 vol% NH_3_ (balanced by He). The gas hourly space velocity (GHSV) was adjusted to 36 L g_cat_^−1^h^−1^ by controlling the flow rate. The reaction was then carried out at various temperatures, which was increased stepwise from 275 to 600 ^o^C, and steady state was allowed to reach before the product analysis. To determine the conversions of reactant, a FTIR spectrometer (Nicolet 6700, Thermo Scientific) equipped with a long path (5 m) gas cell and a MCT detector (with a resolution of 8 cm^−1^) was used to analyze NH_3_ (964 cm^−1^, 929 cm^−1^). The NH_3_ conversion were calculated using ${\mathrm{NH}}_{\mathrm{3}}\,{\mathrm{conversion}} = \frac{{[{\mathrm{NH}}_{\mathrm{3}}]_{{\mathrm{inlet}}} - [{\mathrm{NH}}_{\mathrm{3}}]_{{\mathrm{outlet}}}}}{{\left( {{\mathrm{1 + }}[{\mathrm{NH}}_{\mathrm{3}}]_{{\mathrm{outlet}}}} \right) \times \left[ {{\mathrm{NH}}_{\mathrm{3}}} \right]_{{\mathrm{inlet}}}}} \times {\mathrm{100\% }}$, where [NH_3_]_inlet_ and [NH_3_]_outlet_ refer to the measured concentrations of NH_3_ fed into and flowing out of the reactor. N_2_ and H_2_ were detected by using a GC-BID equipped with Poropak Q packed column with Helium as the carrier gas.

The measurements of reaction rates and kinetics were carried out at reduced catalyst loadings and the GHSV was adjusted to 100 L g_cat_^−1^ h^−1^, ensuring that the reaction condition was within the kinetic zone (<15% conversion) and mass transfer was not limited. The reaction orders were measured at 425 ^o^C. The TOFs (turnover frequencies) were calculated based on the surface metal atoms. The surface metal atoms of bimetallic Co-Mo and HEA-Co_x_Mo_y_ catalysts were estimated by assuming the nanoparticles as perfect FCC cubo-octahedral particles. From the TEM picture of our particle, we measured the lattice parameter (*a*) of the FCC lattice to be 3.776 Å. According to the literature^52^, the number of total atoms (*N*_T_) and the number of surface atoms (*N*_S_) in a perfect fcc cuboctahedral particle vary, respectively, as a function of ${\mathrm{16}}m^3 - 33 m^2 + 24m - 6$ and ${\mathrm{30}}m^2 - 60m + 32$ with variable *m* which is the number of atoms lying on each equivalent edge of the particle. Thus, the diameter of a sphere with a volume equal to *N*_T_ atoms can be expressed as $d = \root {3} \of {{\frac{3}{{{\mathrm{2\pi }}}}N_T}} \cdot a$. Consequently, we estimated that a 21.8 nm particle would contain a total of 403,014 atoms (correspondingly, *m* = 30) in which 25,232 atoms are on the surface, leading to a surface/volume ratio of about 6.26%. The surface metal atoms of Ru catalysts were obtained by referring the literature^26^.

### Modeling and simulation

The thermodynamic properties of HEA-Co_x_Mo_y_ are also checked by the calculations. Taking HEA-Co_25_Mo_45_ as the example, we applied the established procedures to calculate the thermodynamic properties of HEA-Co_25_Mo_45_ alloy based on available empirical data of the atomic size of constituents, formation enthalpy of binary alloy combinations, configuration entropy of the ideal solutions. Specifically, we evaluated a parameter (*δ*) gauging the atomic size difference in the alloys as $\delta = \sqrt {\mathop {\sum }\nolimits_{i{\mathrm{ = 1}}}^n C_i({\mathrm{1 - }}\frac{{r_i}}{{\bar r}})}$ (*C*_*i*_ is the atomic percentage of the *i*th component, *r*_*i*_ is the atomic radius of the *i*th component which can be obtained in the reference 53, $\bar r = \mathop {\sum}\nolimits_{i = 1}^n {C_ir_i}$ is the average atomic radius), the enthalpy of mixing of the alloy as ${\Delta} H_{{\mathrm{mix}}} = \mathop {\sum}_{{i = 1,j {> } i}}^n {\Omega} _{ij} C_{i} C_{j}$ (${\Omega}_{ij} = 4{\Delta}H_{{\mathrm{AB}}}^{{\mathrm{mix}}}$ is the regular solution interaction parameter between the ith and jth elements, ${\Delta} H_{AB}^{{\mathrm{mix}}}$ is the enthalpy of mixing of binary liquid alloys which are obtained in the reference 54, and the entropy of mixing of the n-element regular solution ${\Delta} S_{{\mathrm{mix}}} = - R\mathop {\sum}\nolimits_{i = 1}^n {C_i{\mathrm{ln}}C_i}$(*R* is gas constant).

From the survey of those bulk multi-component alloys to form solid-solution HEAs, it has been found that the formation of HEA most often requires: $\delta \le 6.6\%$ and ${\mathrm{ - 11}}.6 \,\, < \,\,{\Delta} H_{{\mathrm{mix}}} \,\, < \,\, 3 {\mathrm{.2}}({\mathrm{kJ}}\,{\mathrm{mol}}^{{\mathrm{ - 1}}})$^45,46^. In this work, we have calculated that HEA-Co_25_Mo_45_ has ${\mathrm{\delta = 5}}{\mathrm{.4\% }}$, ${\Delta} H_{{\mathrm{mix}}} = {\mathrm{0}}{\mathrm{.65}}({\mathrm{kJ}}\,{\mathrm{mol}}^{{\mathrm{ - 1}}})$, and ${\Delta} S_{{\mathrm{mix}}} = {\mathrm{11}}{\mathrm{.6}}\left( {\left. {{\mathrm{J}}\left( {{\mathrm{K}}\,{\mathrm{mol}}} \right)^{{\mathrm{ - 1}}}} \right)} \right)$. Hence, we predict that HEA-Co_25_Mo_45_ should form solid-solution HEA particles. The atomic interaction energy of HEA-Co_*x*_Mo_*y*_ system is evaluated within the framework of the second nearest neighbor Modified Embedded Atom Method (MEAM)^55,56^. The parameters of the MEAM potentials for pure metal Fe, Co, Ni, Cu, and Mo are taken from references^57–59^. The cross potentials between different metals are developed by fitting the MEAM predictions of the enthalpy of formation and the lattice constants of binary and quaternary alloys against the predictions from the density functional theory (DFT) calculations. All the DFT calculations are carried out using the plane wave basis set and projector augmented wave method and Perdew-Burke-Ernzerhof (PBE) exchange-correlation functional as implemented in the Vienna Ab-initio Simulation Package (VASP)^60–62^. The energy cutoff is set to 500 eV for the plane wave expansion, and the Brillouin zone is sampled by a Monkhorst-Pack k-point mesh of 11 × 11 × 11 for the alloy systems^63^. As presented in Table S1, the MEAM predictions reproduce well the DFT calculation results.

A Monte Carlo (MC) simulation method based on the Metropolis algorithm^64^ is employed to sample the atomic configurations of HEA-Co_*x*_Mo_*y*_ nanoparticle in canonical ensemble^65,66^. Starting from a random atomic configuration, a series of MC trial steps are attempted by performing either of the following two configuration changes: (1) Small displacement of a randomly selects atom along a randomly selected direction, which accounts for the relaxation and local atomic vibration processes. The magnitude of the small displacement is in the range of (0, 0.02Å). (2) Swapping the position of two atoms with different element types, which represents the ling-range diffusion process in the modeled system.

At given temperature *T*, the probability (*p*) to retain the trial configuration is calculated according to Boltzmann distribution with $p = {\mathrm{min}}\left[ {{\mathrm{1,exp}}\left( {{\mathrm{ - }}\frac{{\Delta E}}{{k_BT}}} \right)} \right]$, where ∆*E* is the total energy change of the alloy system, due to the configuration variation, calculated from the MEAM potentials and *k*_*B*_ is the Boltzmann constant.

In each MC simulation, 10 million MC iterations are performed to evaluate the atomic distribution of the nanoparticles, in which the probability of vibrational trial steps (operation 1) is set to 99.9% and the rest steps (0.1%) were assigned to the long-range diffusion operation (operation 2). Thus, we are modeling the equilibrium structures of alloy particles under the situation in which long-range diffusion processes are dynamically slow and limited.

## Supplementary information

Supplementary Information

## Data Availability

The data that support the figures in this work and other findings corresponding to this study are available from the corresponding authors upon reasonable request.
