# Supplementary material for: Highly efficient decomposition of ammonia using high-entropy alloy catalysts
Source: Nat Commun. 2019 Sep 5;10:4011. doi: 10.1038/s41467-019-11848-9 (PMC6728353; doi:10.1038/s41467-019-11848-9)
Supplement: Supplementary file 1 — Supplementary Information [file 41467_2019_11848_MOESM1_ESM.pdf]

Supplementary Information

Highly Efficient Decomposition of Ammonia Using High-Entropy Alloy Catalysts

Pengfei Xie,[1,†] Yonggang Yao,[2,†] Zhenn\an Huang,[3,†] Zhenyu Liu,[4,†] Junlei Zhang,[1] Tangyuan Li,[2] Guofeng Wang,[4,*] Reza Shahbazian-Yassar,[3,*] Liangbing Hu,[2,*] Chao Wang[1,*]

[1] Department of Chemical and Biomolecular Engineering, Johns Hopkins University, Baltimore, Maryland, 21218, USA;

[2] Department of Materials Science and Engineering, University of Maryland, College Park, Maryland, 20742, USA;

[3] Department of Mechanical and Industrial Engineering, University of Illinois, Chicago, Illinois 60607, USA;

[4] Department of Mechanical Engineering & Materials Science, University of Pittsburgh, Pittsburgh, Pennsylvania, 15261, USA.

[†] These authors contributed equally to this work.

* Emails: chaowang@jhu.edu; binghu@umd.edu; rsyassar@uic.edu; guw8@pitt.edu.

Supplementary Tables and Figures

Supplementary Table 1. List of enthalpy of formation and lattice constant for the binary and quaternary alloys predicted by MEAM and DFT calculations.

Alloy	phase	Lattice Constant (Å)		Enthalpy of Formation (eV atom^{-1})	
		DFT	MEAM	DFT	MEAM
Co_3Mo	$L1_2$	3.590	3.658	0.009	0.009
CoMo	B2	2.997	2.997	0.297	0.297
$CoMo_3$	$L1_2$	3.860	3.859	0.210	0.215
Co_3Fe	$L1_2$	3.546	3.490	0.012	0.033
CoFe	B2	2.845	2.843	-0.056	-0.054
$CoFe_3$	$L1_2$	3.484	3.491	0.140	0.091
Co_3Ni	$L1_2$	3.522	3.542	0.003	0.005
CoNi	B2	2.811	2.878	0.156	0.134
$CoNi_3$	$L1_2$	3.525	3.526	0.013	0.014
Co_3Cu	$L1_2$	3.555	3.496	0.130	0.131
CoCu	B2	2.843	2.843	0.268	0.267
$CoCu_3$	$L1_2$	3.612	3.471	0.181	0.183
Mo_3Fe	$L1_2$	3.888	3.801	0.284	0.289
MoFe	B2	3.004	3.004	0.182	0.182
$MoFe_3$	$L1_2$	3.606	3.617	0.095	0.087
Mo_3Ni	$L1_2$	3.864	3.884	0.111	0.106
MoNi	B2	2.991	2.993	0.298	0.305
$MoNi_3$	$L1_2$	3.645	3.614	0.096	0.097
Mo_3Cu	$L1_2$	3.887	3.902	0.209	0.207
MoCu	B2	3.029	3.029	0.522	0.522
$MoCu_3$	$L1_2$	3.766	3.580	0.498	0.492
Fe_3Ni	$L1_2$	3.599	3.541	0.043	0.044
FeNi	B2	2.855	2.846	0.076	0.073
$FeNi_3$	$L1_2$	3.548	3.506	-0.089	-0.089
Fe_3Cu	$L1_2$	3.617	3.574	0.129	0.128
FeCu	B2	2.895	2.895	0.268	0.268
$FeCu_3$	$L1_2$	3.653	3.486	0.219	0.220
Ni_3Cu	$L1_2$	3.549	3.485	0.032	0.032
NiCu	B2	2.841	2.842	0.081	0.081
$NiCu_3$	$L1_2$	3.603	3.489	0.012	0.012
CoMoFeNi	fcc	3.608	3.582	0.038	0.029
CoMoFeCu	fcc	3.642	3.604	0.238	0.146
CoFeNiCu	fcc	3.580	3.513	0.058	0.076
CoMoNiCu	fcc	3.655	3.584	0.257	0.175
MoFeNiCu	fcc	3.649	3.599	0.237	0.135

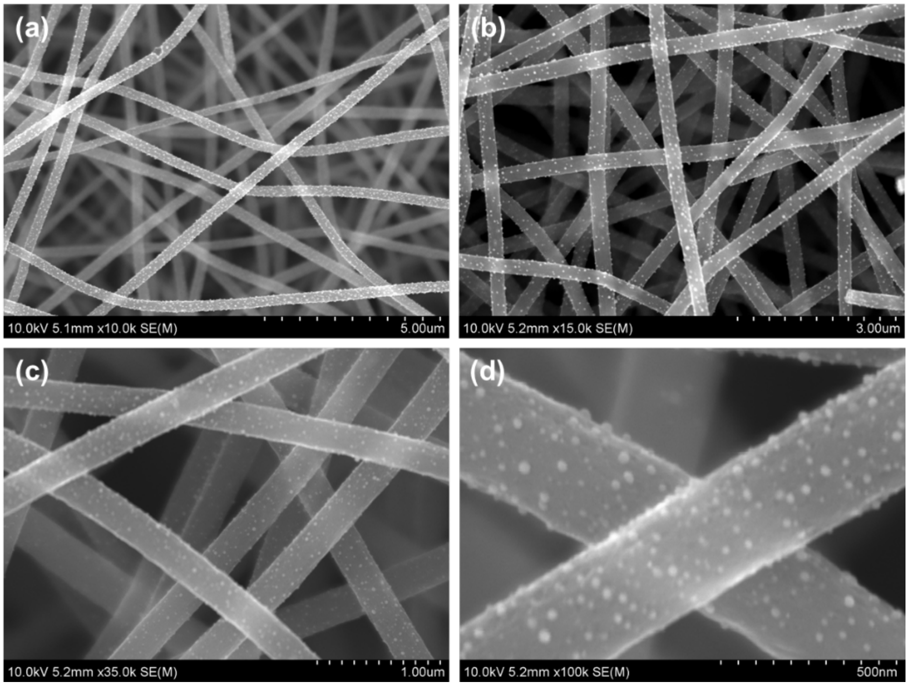

Supplementary Figure 1. SEM images of $Co_{25}Mo_{45}Fe_{10}Co_{10}Ni_{10}$ supported on carbon nanofibers, verifying the dispersion of nanoparticles.

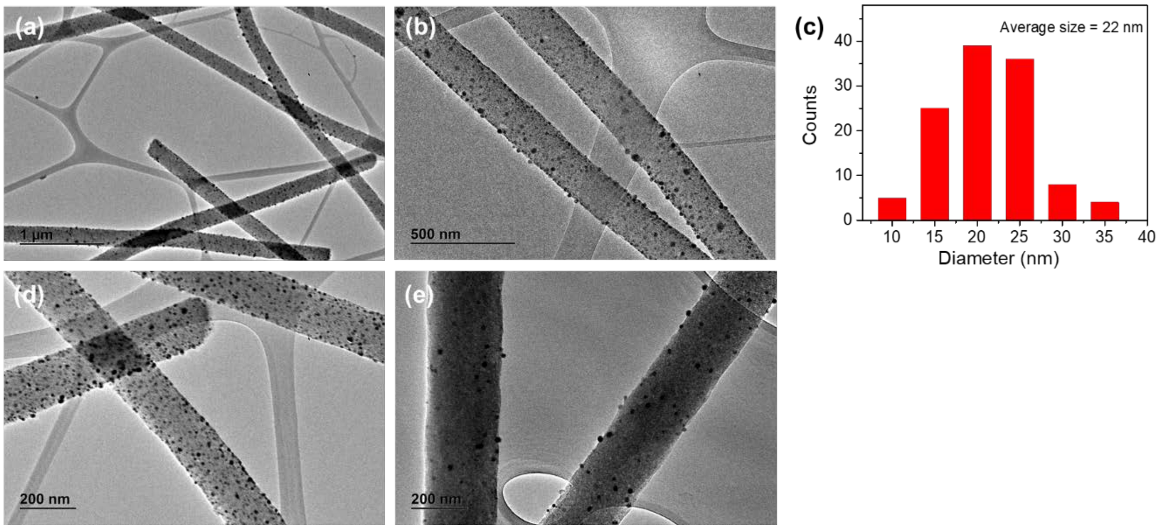

Supplementary Figure 2. TEM images of the $Co_{25}Mo_{45}Fe_{10}Co_{10}Ni_{10}$ nanoparticles supported on CNFs.

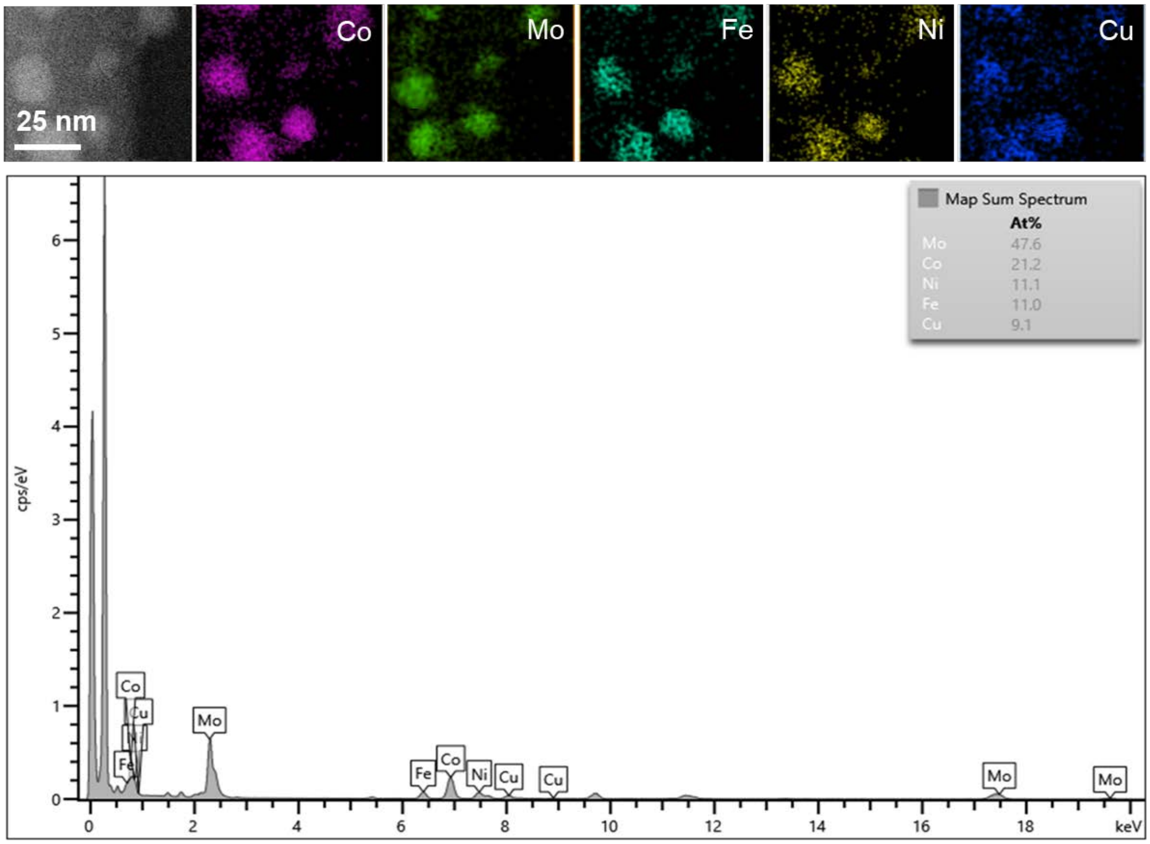

Supplementary Figure 3. STEM elemental maps (a) and corresponding spectrum at a low magnification for $Co_{25}Mo_{45}Fe_{10}Co_{10}Ni_{10}$ supported on CNF.

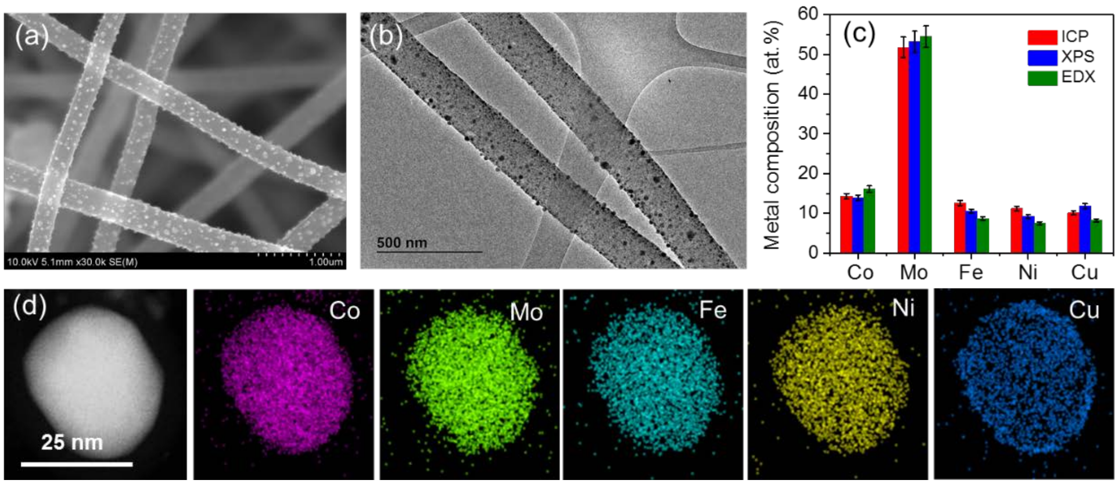

Supplementary Figure 4. SEM (a) and TEM (b) images of $Co_{15}Mo_{55}Fe_{10}Co_{10}Ni_{10}$ nanoparticles well dispersed on CNFs. (c) Comparison of the composition analyses for $Co_{15}Mo_{55}Fe_{10}Co_{10}Ni_{10}$ using three different techniques (ICP-MS, XPS, and EDX), verifying the homogeneous alloy nature. Error bars denote SDs. (d) STEM elemental maps of $Co_{15}Mo_{55}Fe_{10}Co_{10}Ni_{10}$ nanoparticles.

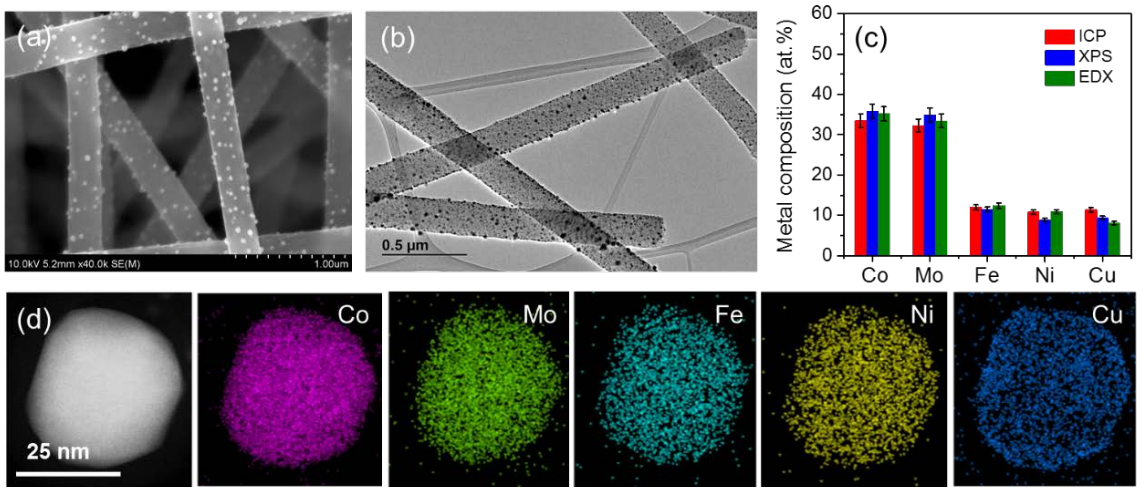

Supplementary Figure 5. SEM (a) and TEM (b) images of $Co_{35}Mo_{35}Fe_{10}Co_{10}Ni_{10}$ nanoparticles well dispersed on CNFs. (c) Comparison of the composition analyses for $Co_{35}Mo_{35}Fe_{10}Co_{10}Ni_{10}$ using three different techniques (ICP-MS, XPS, and EDX), verifying the homogeneous alloy nature. Error bars denote SDs. (d) STEM elemental maps of $Co_{35}Mo_{35}Fe_{10}Co_{10}Ni_{10}$ nanoparticles.

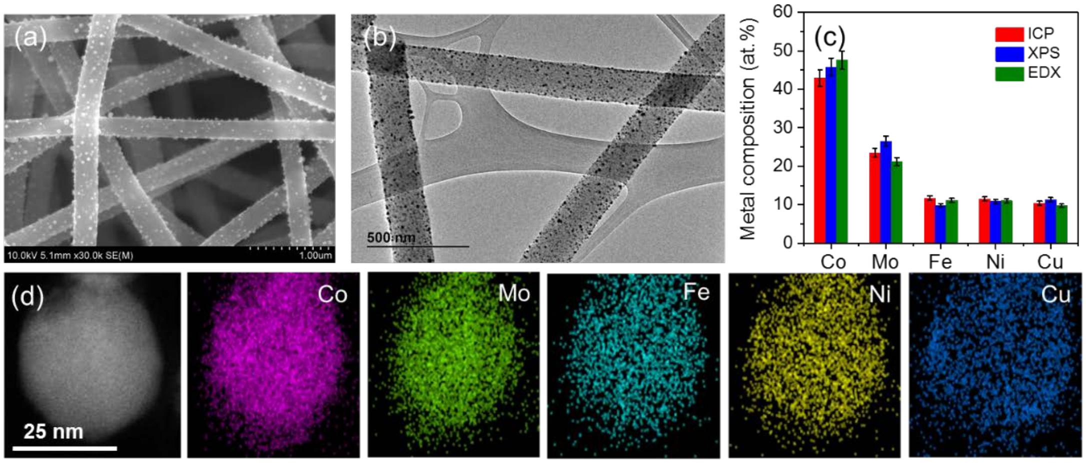

Supplementary Figure 6. SEM (a) and TEM (b) images of $Co_{45}Mo_{25}Fe_{10}Co_{10}Ni_{10}$ nanoparticles well dispersed on CNFs. (c) Comparison of the composition analyses for $Co_{45}Mo_{25}Fe_{10}Co_{10}Ni_{10}$ using three different techniques (ICP-MS, XPS, and EDX), verifying the homogeneous alloy nature. Error bars denote SDs. (d) STEM elemental maps of $Co_{45}Mo_{25}Fe_{10}Co_{10}Ni_{10}$ nanoparticles.

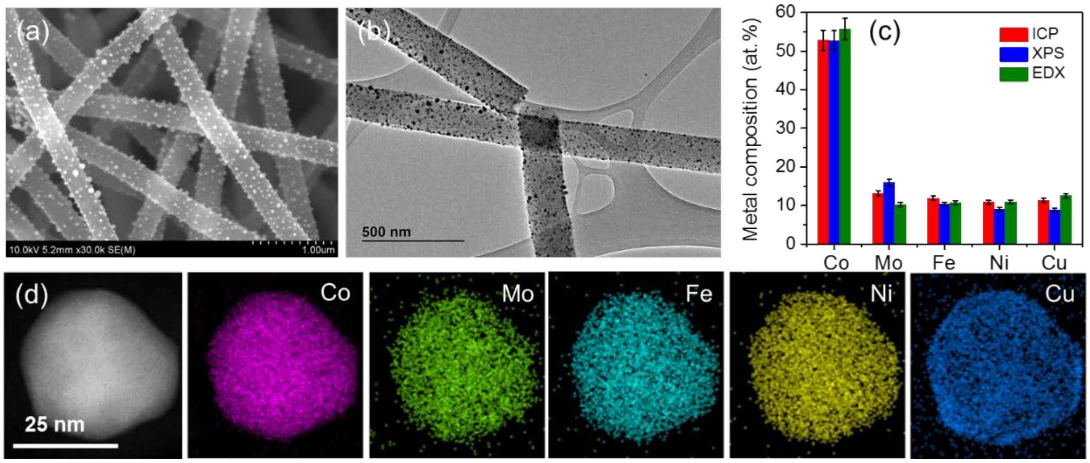

Supplementary Figure 7. SEM (a) and TEM (b) images of $Co_{55}Mo_{15}Fe_{10}Co_{10}Ni_{10}$ nanoparticles well dispersed on CNFs. (c) Comparison of the composition analyses for $Co_{55}Mo_{15}Fe_{10}Co_{10}Ni_{10}$ using three different techniques (ICP-MS, XPS, and EDX), verifying the homogeneous alloy nature. Error bars denote SDs. (d) STEM elemental maps of $Co_{55}Mo_{15}Fe_{10}Co_{10}Ni_{10}$ nanoparticles.

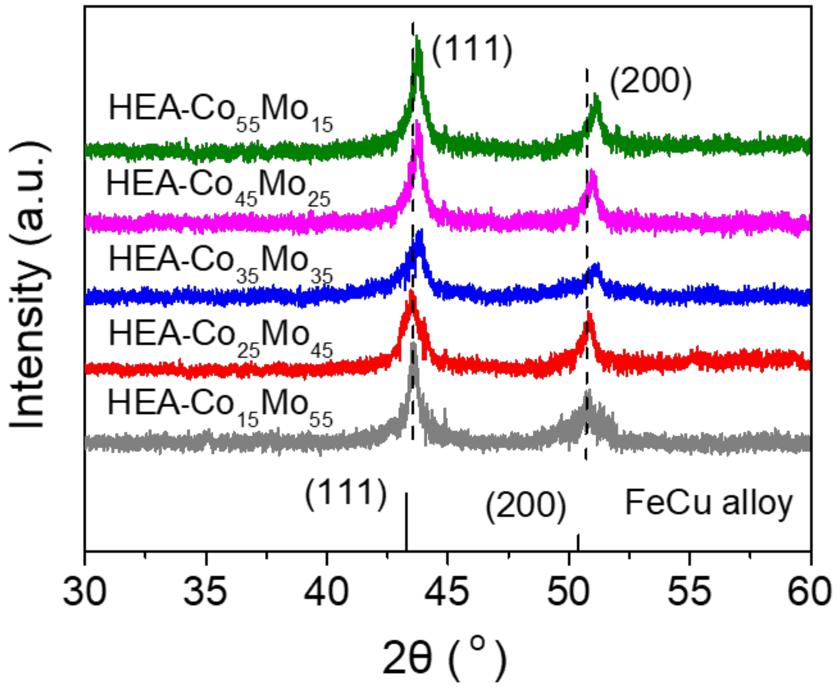

Supplementary Figure 8. Comparison of the XRD patterns of HEA-Co_xMo_y catalysts and with the standard patterns of FeCu (JCDPS no. 65-7002).

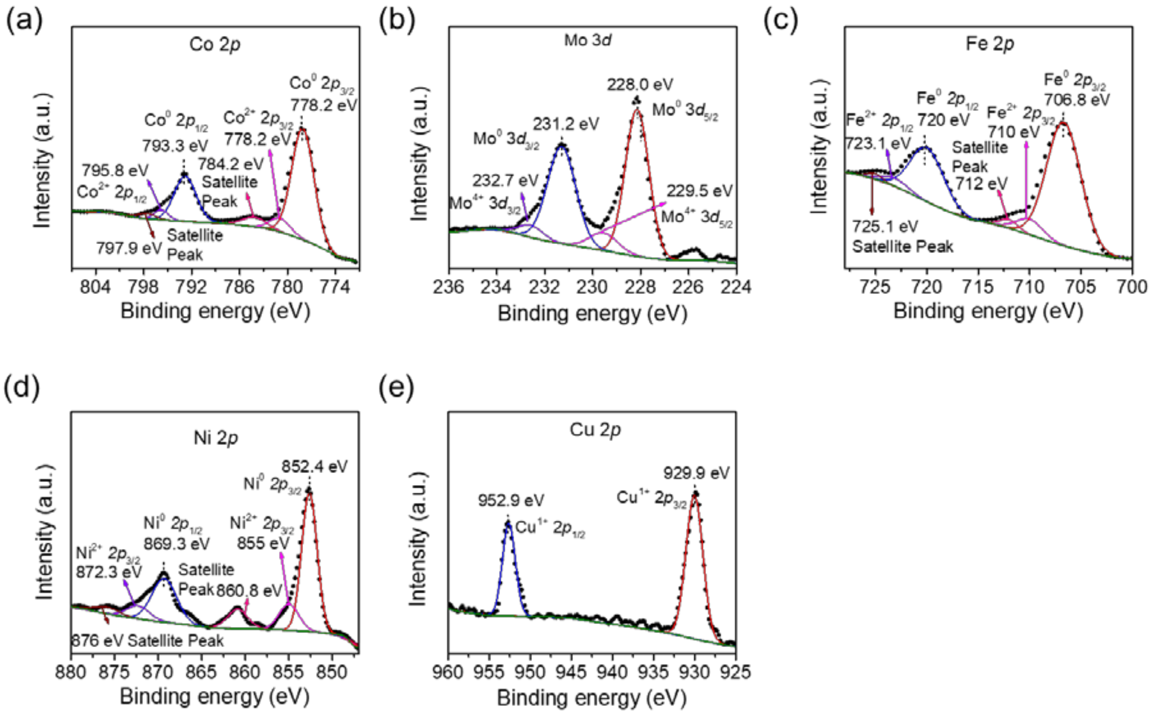

Supplementary Figure 9. (a-e) XPS spectra at the Co 3*d*, Mo 2*p*, Fe 2*p*, Ni 2*p* and Cu 2*p* edges collected on $Co_{25}Mo_{45}Fe_{10}Co_{10}Ni_{10}$ nanoparticles after the NH_3 decomposition reaction.

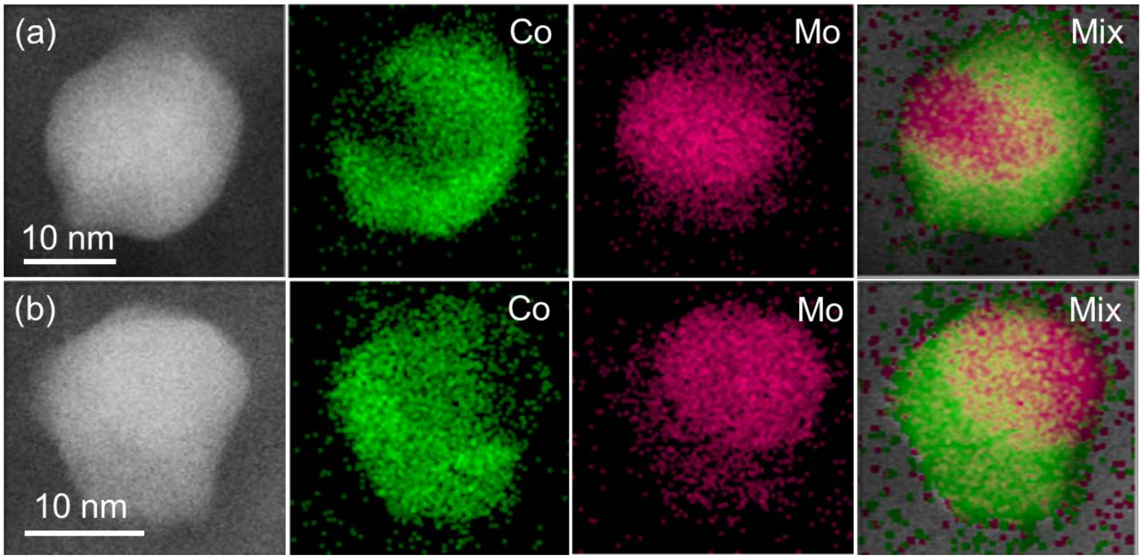

Supplementary Figure 10. STEM elemental maps of bimetallic CoMo alloy nanoparticles (Co/Mo = 25/45), which showing segregated phases and demonstrating immiscibility with the current Co/Mo ratio.

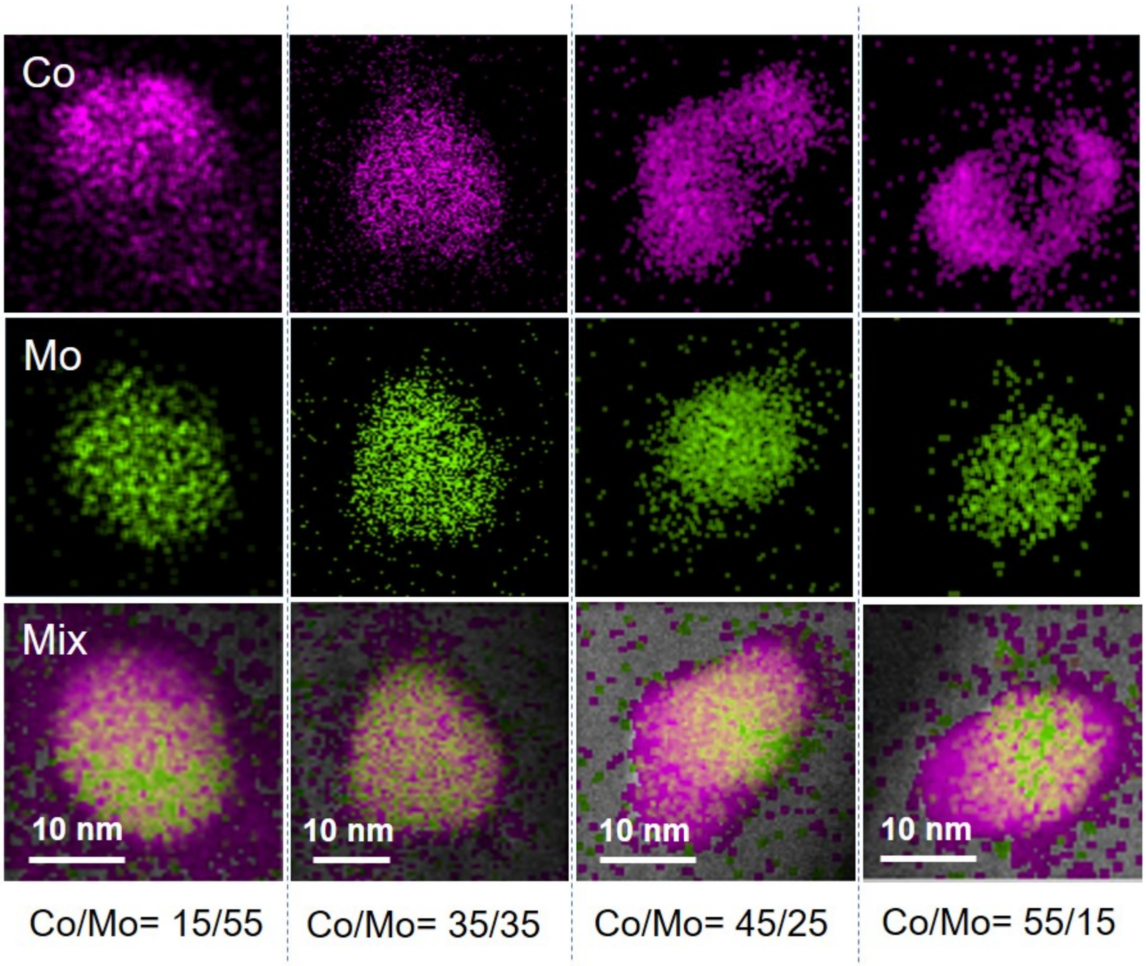

Supplementary Figure 11. STEM elemental maps of Bimetallic CoMo alloy nanoparticles (Co/Mo = 15/55, 35/35, 45/25 and 55/15), demonstrating that bimetallic CoMo alloy is immiscible except the Co/Mo close to 1 (i.e., bimetallic CoMo, Co/Mo = 35/35).

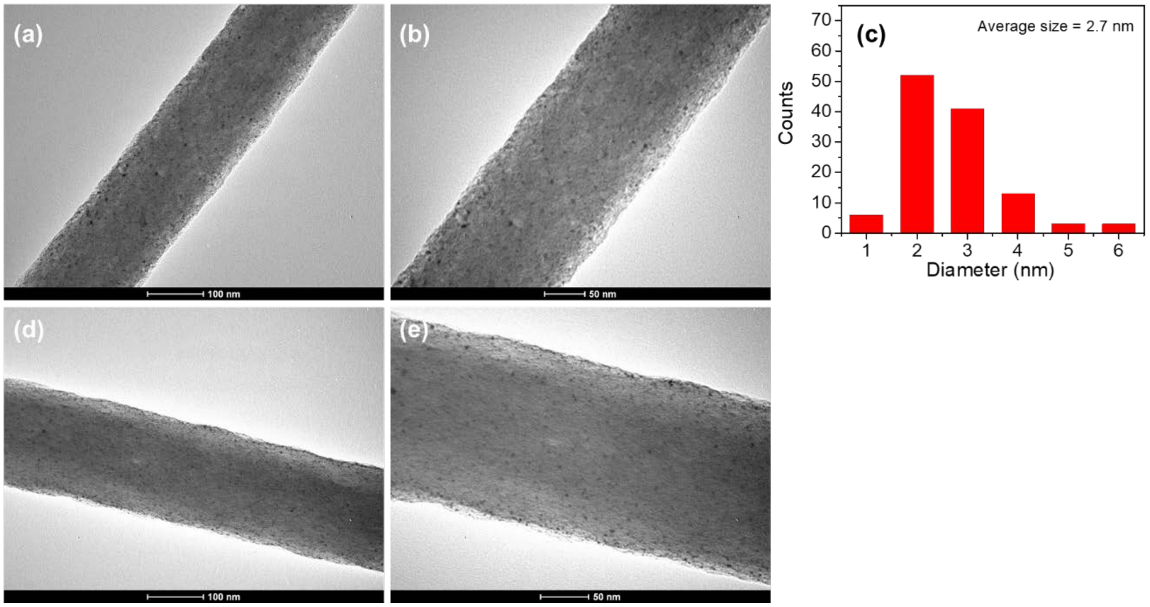

Supplementary Figure 12. TEM images of Ru supported on carbon nanofibers (CNF), showing the average size of nanoparticles is 2.7 nm.

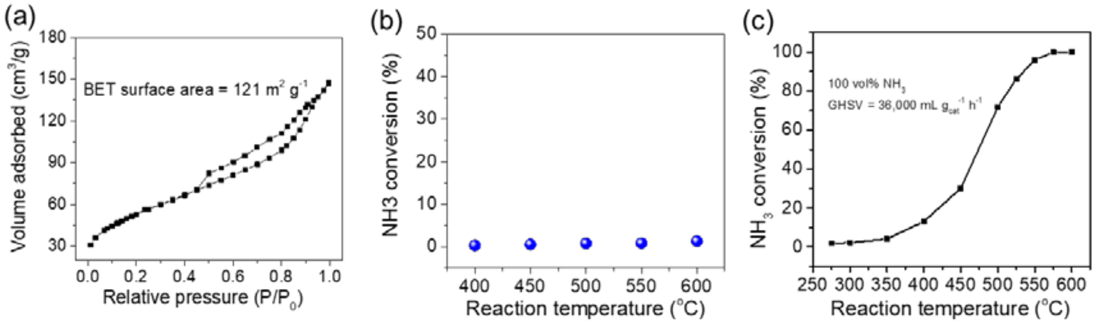

Supplementary Figure 13. Characterization and catalytic performance of bare carbon nanofibers. (a) N_2 adsorption and desorption isotherms and (b) NH_3 decomposition measured on the bare CNF substrates. Reaction condition in (b): 5 vol% NH_3, space velocity = 36 L g_{cata}^{-1} h^{-1}. (c) NH_3 decomposition measured on the Ru/CNF substrates with 100 vol% NH_3, space velocity = 36 L g_{cata}^{-1} h^{-1}.

Supplementary Table 2. Comparison of our Ru/CNFs benchmark with the Ru catalysts reported in the literature for decomposition of 100 vol% NH_3.

Catalysts	T<br>°C	GHSV<br>mL g_{cat}^{-1} h^{-1}	NH_3 Conversion<br>%	Ref
Ru/CNTs	500	30,000	84	1
Ru/Graphene	500	20,000	82	2
Ru/amorphous carbon	550	30,000	14	3
Ru/TiO_2	500	30,000	64	4
Ru/Al_2O_3	500	30,000	58	5
Ru/CNFs	500	36,000	72	This work
HEA-$Co_{55}Mo_{15}$	500	36,000	100	This work

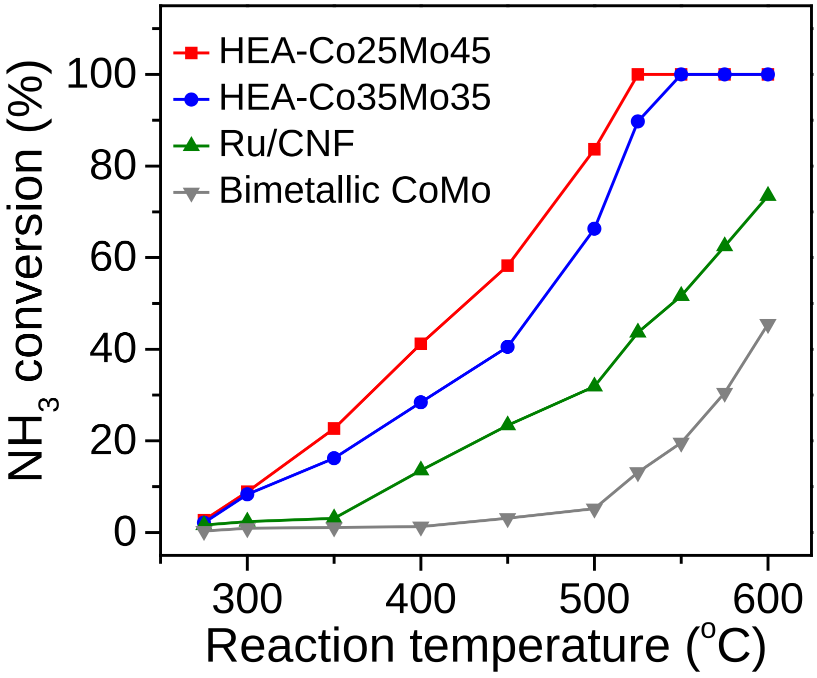

Supplementary Figure 14. NH_3 conversions depended on reaction temperatures over HEA-Co_xMo_y nanoparticles, Ru/CNF, and bimetallic CoMo (Co/Mo = 25/45) Reaction condition: 5 vol% NH_3, Space velocity = 36 L g_{cata}^{-1} h^{-1}.

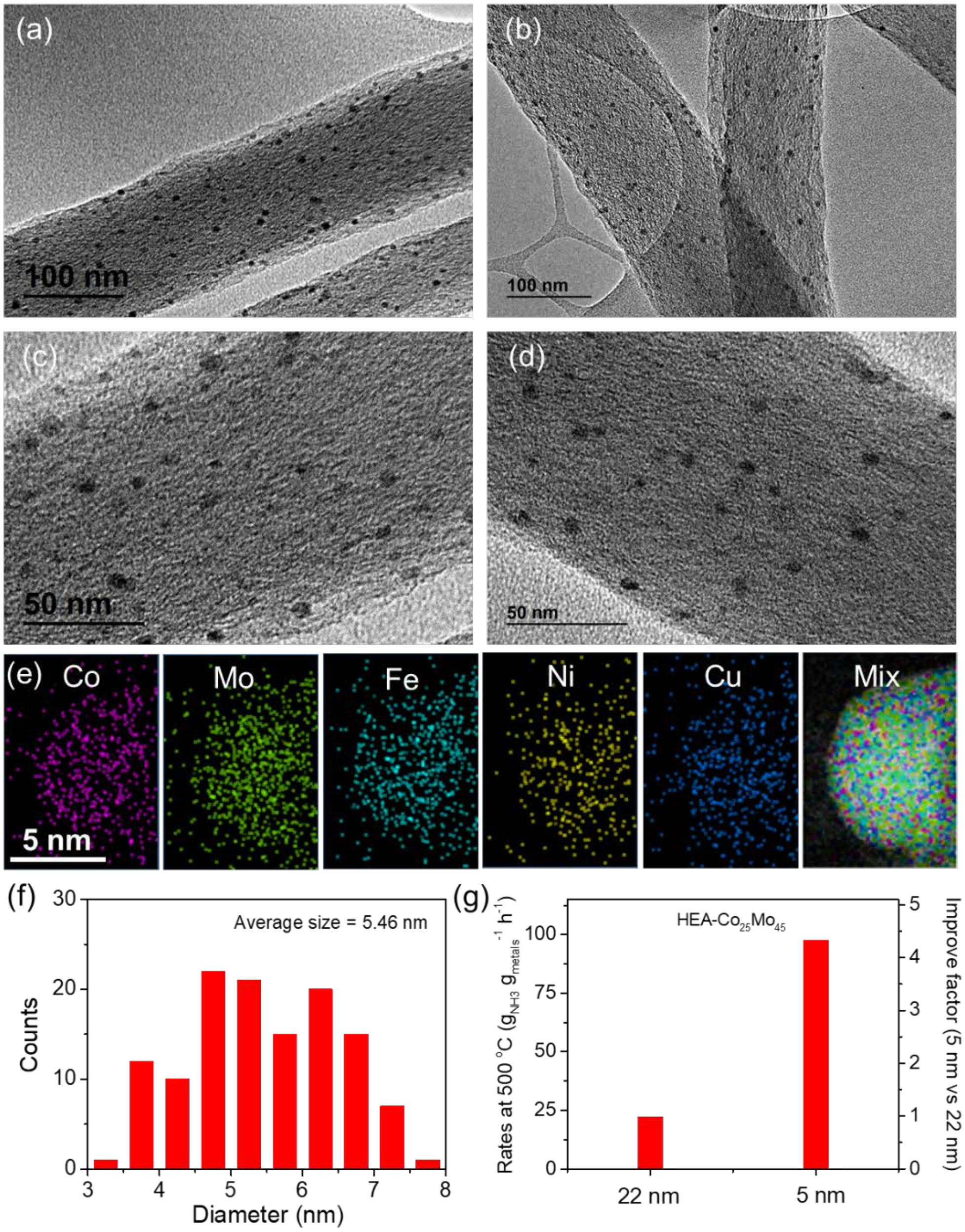

Supplementary Figure 15. (a-d) TEM images of $Co_{25}Mo_{45}Fe_{10}Co_{10}Ni_{10}$-S (denoted as HEA-$Co_{25}Mo_{45}$-S) supported on CNFs. (e) STEM-based elemental maps of the HEA-$Co_{25}Mo_{45}$-S nanoparticles (f) Particle distribution of HEA-$Co_{25}Mo_{45}$-S nanoparticles showing the nanoparticle sizes are in the range of 3-8 nm and the average size of ~5 nm. (g) Comparison of reaction rates measured in the kinetic regime among HEA-$Co_{25}Mo_{45}$-S and HEA-$Co_{25}Mo_{45}$ (T = 500 ^{o}C).

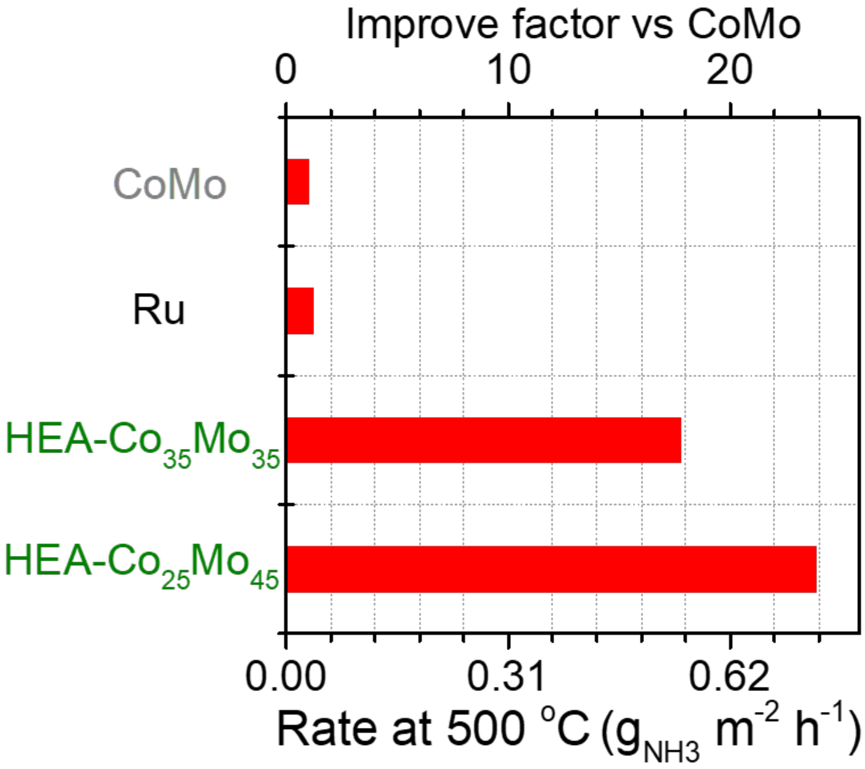

Supplementary Figure 16. Comparison of reaction rates normalized by specific surface areas (estimated from the average particle sizes) between bimetallic Co-Mo, Ru, and Co_xMo_y-HEA catalysts (T = 500 °C, 5 vol% NH_3, GHSV = 36 L_{NH3} g_{cata}^{-1} h^{-1}).

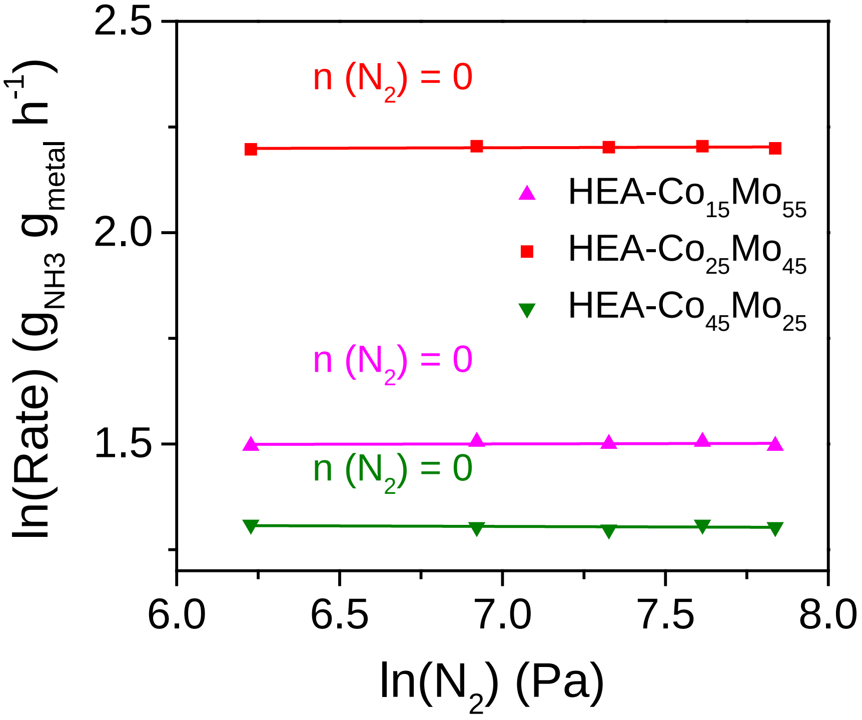

Supplementary Figure 17. The Reaction orders of N_2 during NH_3 decomposition over HEA-Co_xMo_y catalysts.

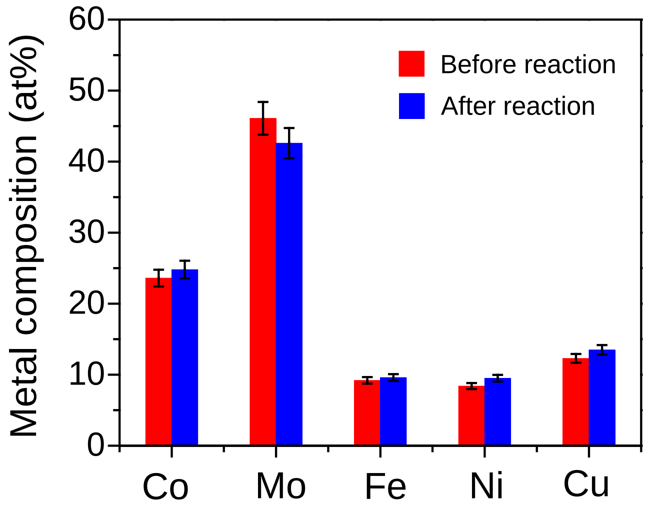

Supplementary Figure 18. Composition analysis by XPS of $Co_{25}Mo_{45}Fe_{10}Co_{10}Ni_{10}$ nanoparticles before and after NH_3 decomposition. Error bars denote SDs.

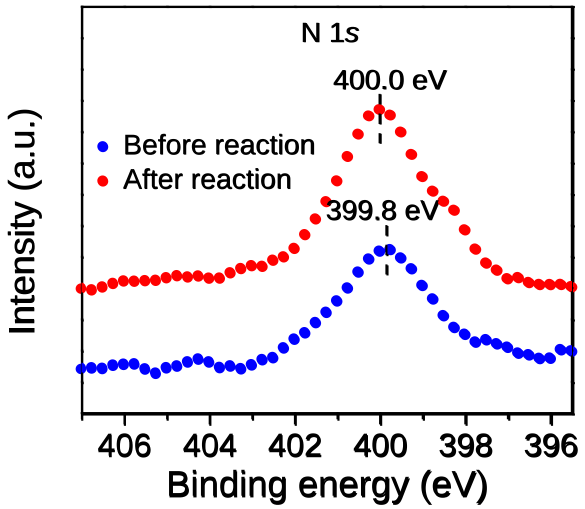

Supplementary Figure 19. Comparison of the weak N 1s XPS peak of HEA-$Co_{25}Mo_{45}$ before and after the reaction.

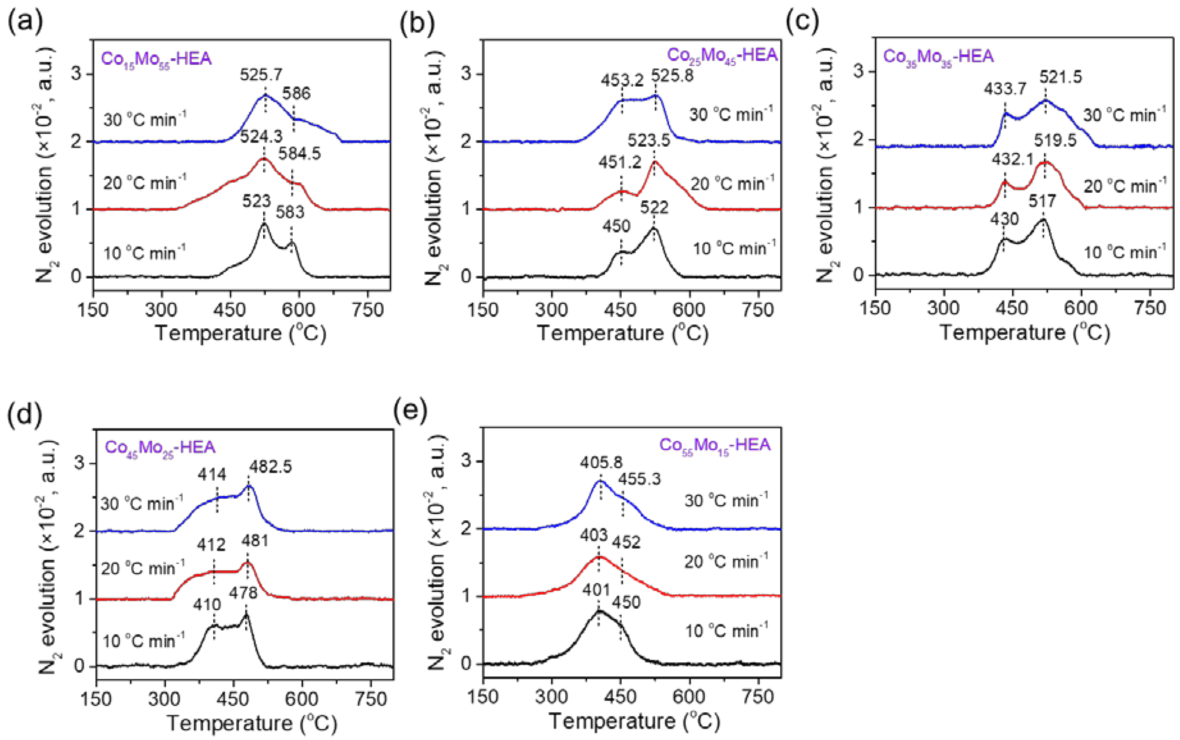

Supplementary Figure 20. Nitrogen TPD profiles recorded at different ramping rates on (a) HEA-$Co_{15}Mo_{55}$, (b) HEA-$Co_{25}Mo_{45}$, (c) HEA-$Co_{35}Mo_{35}$, (d) HEA-$Co_{45}Mo_{25}$ and (e) HEA-$Co_{55}Mo_{15}$ catalysts.

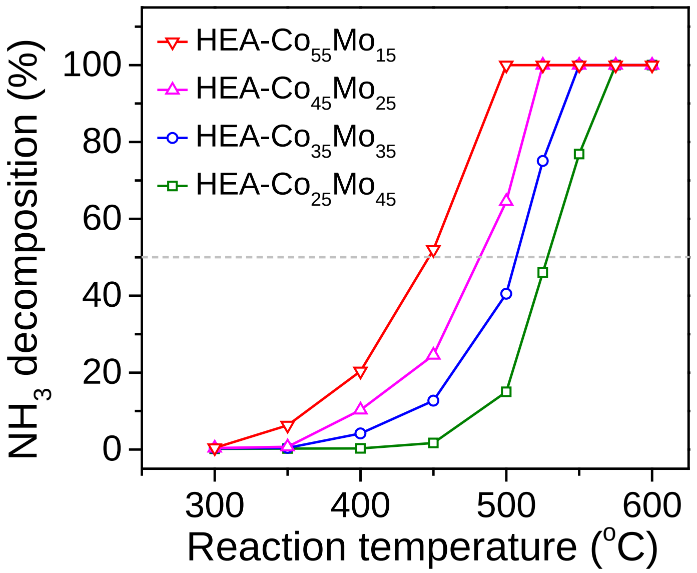

Supplementary Figure 21. Catalytic performance of NH_3 decomposition over HEA-Co_xMo_y nanoparticles supported CNF. Reaction condition: 100 vol% NH_3, Space velocity = 36 L g_{cata}^{-1} h^{-1}.

Supplementary Reference

1 Yin, S. F., Xu, B. Q., Zhou, X. P. & Au, C. T. A mini-review on ammonia decomposition catalysts for on-site generation of hydrogen for fuel cell applications. *Appl Catal a-Gen* **277**, 1-9 (2004).

2 Li, G., Kanezashi, M. & Tsuru, T. Catalytic ammonia decomposition over high-performance Ru/Graphene nanocomposites for efficient COx-free hydrogen production. *Catalysts* **7** (2017).

3 Li, L., Zhu, Z. H., Yan, Z. F., Lu, G. Q. & Rintoul, L. Catalytic ammonia decomposition over Ru/carbon catalysts: The importance of the structure of carbon support. *Appl Catal a-Gen* **320**, 166-172 (2007).

4 Yin, S. F., Xu, B. Q., Zhu, W.X., Ng, C.F., Zhou, X. P. & Au, C. T. Carbon nanotubes-supported Ru catalyst for the generation of COx-free hydrogen from ammonia. *Catal Today* **93-5**, 27-38 (2004).

5 Karim, A. M., Prasad, V., Mpourmpakis, G., Lonergan, W.W, Frenkel, A.I., Chen J.G., & Vlachos D.G. Correlating particle size and shape ofsupported Ru/gamma-Al_2O_3 catalysts with NH_3 decomposition activity. *J Am Chem Soc* **131**, 12230-12239 (2009).
